# Iron-Related Metabolic Targets in the Treatment of Osteosarcoma: Research Progress and Prospects

**DOI:** 10.3390/biomedicines13112756

**Published:** 2025-11-11

**Authors:** Arianna Buglione, Magda Gioia, Federica Sinibaldi, Stefano Marini, Chiara Ciaccio

**Affiliations:** Department of Clinical Sciences and Translational Medicine, University of Rome ‘Tor Vergata’, Via Montpellier 1, I-00133 Rome, Italy; arianna.buglione@alumni.uniroma2.eu (A.B.); magda.gioia@uniroma2.it (M.G.); federica.sinibaldi@uniroma2.it (F.S.); stefano.marini@uniroma2.it (S.M.)

**Keywords:** osteosarcoma, iron metabolism, ferroptosis, ferritinophagy, ferroptosis-related ncRNA networks, nanomedicine, targeted therapy, drug resistance

## Abstract

Iron metabolism has emerged as a critical regulator of cancer biology, with mounting evidence linking iron dysregulation to tumor initiation, progression, and resistance mechanisms. Osteosarcoma (OS) is the most common primary bone malignancy and a leading cause of cancer-related death in children and young adults; recent studies have identified profound alterations in iron homeostasis at both cellular and microenvironmental levels in OS. These include increased iron uptake, disrupted storage and export, and a reliance on iron-dependent metabolic pathways that promote proliferation, metastasis, and immune evasion. Despite advances in surgical and chemotherapeutic approaches, survival outcomes in OS have stagnated, underscoring the need for novel therapeutic strategies. Targeting iron metabolism represents a promising avenue, with strategies such as iron chelation, transferring receptor inhibition, ferroptosis induction, and modulation of ferritinophagy, showing preclinical efficacy. In this review, we provide an updated and integrated overview of the multifaceted role of iron in OS pathogenesis, dissect emerging therapeutic approaches aimed at disrupting iron regulatory networks, and highlight innovative delivery platforms including nanomedicine. By integrating current insights on iron metabolism with the molecular complexity of OS, we present a comprehensive perspective, while acknowledging that the limited clinical translatability of current findings still hinders progress toward clinical application. A deeper understanding of iron-driven mechanisms may guide future studies toward the development of safe and effective iron-targeted therapies for OS.

## 1. Introduction

In recent years, growing attention has been directed toward iron metabolism in cancer biology, marking what many consider a “golden age” of discovery in this field [1,2]. Numerous iron-related proteins and regulatory mechanisms have been identified, expanding our understanding of iron’s physiological roles and its impact on tumorigenesis. There is now compelling evidence linking dysregulated iron metabolism to fundamental oncogenic processes, including tumor initiation, proliferation, metastasis, and adaptation to the tumor microenvironment [2,3,4]. Compared to normal cells, cancer cells display profound alterations in iron homeostasis. These include overexpression or dysregulation of iron-handling proteins, leading to elevated intracellular iron levels and enhanced activity of iron-dependent enzymes involved in DNA synthesis and repair, cell cycle progression, angiogenesis, and epigenetic remodeling [3,4]. Moreover, cancer cells demonstrate remarkable plasticity in managing iron availability, actively modulating iron uptake, storage, and export to sustain their metabolic demands [3,5]. This is further shaped by the tumor microenvironment, where conditions such as hypoxia and chronic inflammation promote the secretion of iron-binding proteins, reinforcing iron availability for tumor growth [3,4,6]. Nevertheless, cancer cells are often functionally iron-poor because of their high iron consumption rates [7]. Iron overload, on the other hand, can contribute to tumor initiation and progression through oxidative stress and DNA damage. These opposing conditions are likely temporally distinct, reflecting different stages of cancer development and metabolic reprogramming [7]. These intricate adaptations underscore the crucial role of iron metabolism in cancer survival and progression. As a result, therapeutic strategies aimed at disrupting iron homeostasis, such as iron chelation, induction of ferroptosis (an iron-dependent form of cell death), and inhibition of iron-regulatory pathways, have demonstrated anticancer efficacy in preclinical models. Despite this potential, iron metabolism remains a relatively underexploited target in oncology [3,4].

Osteosarcoma (OS) is the most common primary malignant tumor of the bone, accounting for approximately 56% of all bone cancers. It arises from osteoid and/or immature bone produced by malignant mesenchymal cells and typically affects children and young adults (mostly 14–18 years of age), a period marked by rapid skeletal growth and active extracellular matrix (ECM) remodeling [8,9]. OS typically develops in the metaphysis of long bones, such as the distal femur and proximal tibia, and is associated with high malignancy, early pulmonary metastasis, and poor therapeutic responsiveness [9].

Despite advances in multimodal treatment, including neoadjuvant and adjuvant chemotherapy, surgical resection, long-term survival outcomes for OS have plateaued since the 1980s [9,10]. This stagnation is mainly attributed to the tumor’s profound heterogeneity, aggressive biological behavior, and intrinsic resistance to conventional chemotherapies [8,9]. Although substantial progress has been made in molecular profiling, leading to the identification of potential therapeutic targets and the classification of OS into molecular subtypes [11,12], the biomechanical properties and metabolic vulnerabilities of OS cells remain underexplored.

Within this context, emerging research has revealed profound dysregulation in iron regulatory networks in OS, at both systemic and cellular levels. This results in intracellular iron accumulation, which contributes to tumor growth, resistance to ferroptosis and other forms of cell death, and metastatic dissemination. The iron-rich tumor microenvironment further facilitates immune evasion and contributes to therapeutic resistance, as extensively reviewed elsewhere [6,13]. Given this iron dependency, OS presents a compelling candidate for iron-targeted therapeutic strategies. These include the use of iron chelators [14], transferrin receptor (TfR1) inhibitors [15,16], and inducers of ferroptosis, a form of iron-dependent non-apoptotic cell death [17]. Additionally, emerging approaches such as the modulation of ferritinophagy an iron metabolism-related processes, and nanomedicine-based delivery systems are being investigated for their ability to selectively disrupt iron homeostasis in OS cells [18,19].

In this review, we provide a comprehensive overview of recent advances in iron metabolism-targeted strategies for OS. We examine the role of iron-regulatory networks in OS progression, the interplay between iron homeostasis and the tumor microenvironment, and the emerging links between iron signaling and epigenetic regulation, highlighting their potential as tumor biomarkers and therapeutic targets. Furthermore, we discuss current and experimental therapeutics, ranging from iron chelators to ferroptosis and ferritinophagy regulators, focusing on their mechanisms of action and potential for integration into combination therapies. By dissecting the intersection of iron biology and OS pathophysiology, we aim to shed light on novel therapeutic opportunities that may enhance clinical outcomes in this challenging malignancy.

## 2. The Relevance of Iron in Cell Biology

Iron is one of the most abundant and essential transition metals in the human body, playing a central role in a wide array of biological processes, ranging from structural to enzymatic [1,20,21,22]. Biological systems have co-opted iron due to its ability to transiently bind gaseous ligands and to efficiently catalyze reduction–oxidation reactions necessary for cellular function [1,23]. The biological relevance of iron stems primarily from its unique redox properties and its ability to cycle between two oxidation states, ferrous (Fe^2+^) and ferric (Fe^3+^), which allows it to participate in electron transfer reactions vital for cellular metabolism. Notably, the ferrous form is more soluble and bioavailable than ferric iron, and the reversible interconversion between these two states is critical for the activity of many cellular proteins [1,23]. However, these same redox properties also render iron potentially toxic, creating a paradox in which iron is both indispensable for life and a potential source of cellular damage if not properly regulated.

Most body iron is incorporated into functional biomolecules serving as metal cofactor for many enzymes, either nonheme iron-containing proteins or hemoproteins. Hemoproteins such as hemoglobin and myoglobin are involved in oxygen transport and storage, while cytochromes, catalases, and peroxidases contribute to electron transfer, detoxification, and oxidative stress responses [1,24,25]. Nonheme iron-containing enzymes are similarly crucial, playing roles in DNA synthesis, repair, and telomere maintenance (e.g., ribonucleotide reductase; Fe–S cluster-dependent DNA polymerases such as Pol δ and Pol ε; and helicases like XPD and RTEL1), mitochondrial energy production (e.g., aconitase and Fe–S proteins of the electron transport chain) [1,22], and the biosynthesis of key molecules like collagen and neurotransmitters [1,26]. Despite these critical functions, iron’s redox chemistry presents a major challenge. In the presence of hydrogen peroxide, ferrous iron catalyzes the Fenton reaction, producing highly reactive hydroxyl radicals (OH•), an aggressive form of reactive oxygen species (ROS) that can damage DNA, lipids, and proteins [1,24,25]. Moreover, at physiological pH, ferric iron readily forms insoluble hydroxide polymers (Fe(OH)_3_), limiting its bioavailability.

As such, excess “free” iron poses a significant cytotoxic threat, contributing to oxidative stress and cell injury [1,24,25]. To manage the opposing demands of ensuring iron availability for essential functions while preventing toxicity, organisms have evolved sophisticated systems for its acquisition, transport, storage, and utilization. These systems maintain adequate iron levels for vital cellular processes while minimizing harmful ROS production [27,28]. This delicate balance highlights iron’s exceptional importance in cellular physiology and its central role in health and disease.

## 3. Iron Homeostasis and Its Regulation in Physiological States

The human body contains an average of 3–5 g of iron (of which 60% are present in hemoglobin). To meet daily iron requirements, about 80–90% of iron is recycled from the breakdown of senescent red blood cells, while only 10–20% is typically obtained from dietary absorption in the intestine [1]. Dietary iron exists in two primary forms: heme and nonheme [1,27]. The absorption of dietary non-heme iron occurs through a series of regulated steps. In the intestinal lumen, ferric iron (Fe^3+^) is first reduced to ferrous iron (Fe^2+^) by duodenal cytochrome b reductase (DCYTB). The ferrous iron is then transported into enterocytes via the divalent metal transporter 1 (DMT1). In contrast, most non-intestinal cells acquire iron through receptor-mediated endocytosis. In addition to non-heme iron absorption, heme iron is taken up by intestinal cells through heme carrier protein 1 (HCP1) [29]. Once inside enterocytes, iron that is not immediately utilized can either be stored in ferritin or exported into the bloodstream via the iron exporter ferroportin (FPN), depending on systemic iron requirements. Before export, iron is oxidized back to its ferric (Fe^3+^) form by the multicopper oxidase hephaestin [1,27,28]. Ferric iron then binds to transferrin (TF), a serum glycoprotein responsible for iron transport. Transferrin-bound ferric iron interacts with transferrin receptor 1 (TFR1) on the cell membrane, forming a complex that is internalized via endocytosis. As the resulting endocytic vesicle matures and acidifies into a lysosome, the low pH environment promotes iron release from transferrin [28,30]. Inside the vesicle, ferric iron is reduced to its ferrous form by metalloreductases such as STEAP3 (Six-Transmembrane Epithelial Antigen of Prostate 3).

The released ferrous iron is then transported into the cytosol via DMT1, contributing to the labile iron pool (LIP), a metabolically active and redox-reactive iron pool that supplies iron to various organelles and enzymes.

To ensure the safe and efficient use of ferrous iron within the cytosol, iron chaperones such as PCBP1 and PCBP2 (Poly(rC)-binding proteins 1 and 2) play a central role. These proteins bind Fe^2+^ and deliver it to target destinations, including ferritin for storage and various iron-dependent enzymes, such as deoxyhypusine hydroxylase and prolyl hydroxylase [30,31]. While their functions overlap, PCBP1 is primarily responsible for ferritin loading and iron-sulfur cluster assembly, whereas PCBP2 facilitates iron transfer from importers like DMT1 and supports export via FPN-1. Together, they help maintain intracellular iron homeostasis, preventing free iron accumulation and minimizing oxidative stress [30,31].

Ferritin is a multimeric protein that assembles into a 24-subunit, cage-like complex composed of two structurally similar but functionally distinct subunits: the light chain (FTL) and the heavy chain (FTH1). Each ferritin oligomer can store up to approximately 4000 ferric iron atoms in its central mineral core in a relatively inert form [30,32]. The LIP can be replenished through ferritin degradation by autophagy, a process known as ferritinophagy, mediated by the cargo receptor nuclear receptor coactivator 4 (NCOA4) [19,32]. Alternatively, FPN, the only known iron exporter, can transport iron out of cells, helping to prevent intracellular iron overload. FPN activity is tightly regulated by hepcidin (HEPC), a peptide hormone predominantly produced by the liver. HEPC binds to ferroportin which is expressed on iron-storing hepatocytes, iron-recycling macrophages, and duodenal enterocytes, inducing its internalization and lysosomal degradation. This HEPC-mediated suppression of FPN leads to decreased iron export and increased intracellular iron accumulation, particularly in enterocytes [28,30].

**Figure 1:**
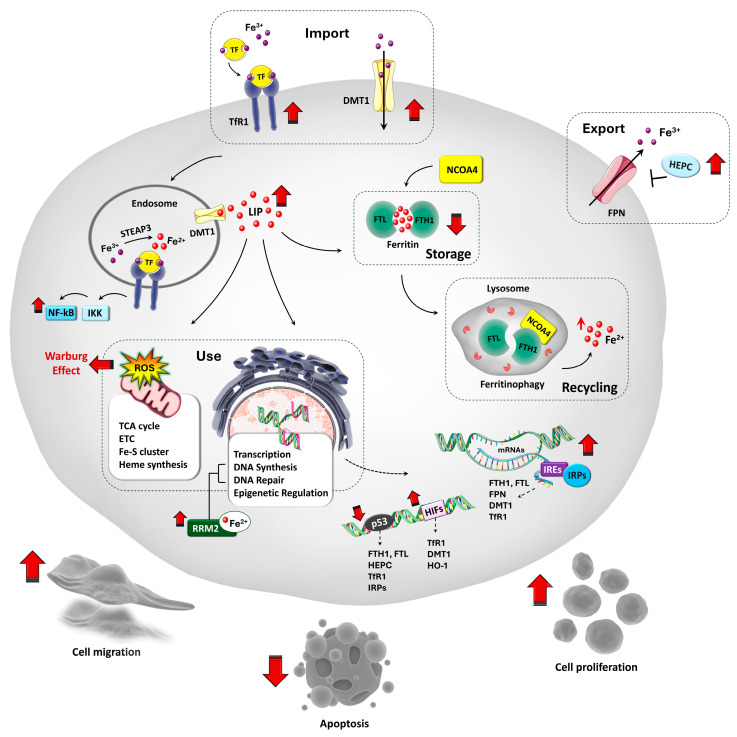
Cellular iron homeostasis and its alteration in OS. Iron is imported primarily through two pathways: via DMT1 or through endocytosis mediated by TfR1 bound to Tf. Within the endosome, Fe^3+^ is released from Tf, reduced to Fe^2+^ by STEAP3, and transported into the cytosol, contributing to the LIP. From the LIP, iron is distributed to intracellular compartments such as mitochondria and the nucleus for metabolic functions, stored in cytoplasmic ferritin (composed of FTL and FTH), or exported by FPN. FPN levels are negatively regulated by HEPC, which induces its internalization and degradation. Iron stored in ferritin can be mobilized through ferritinophagy mediated by NCOA4. Iron homeostasis is tightly regulated by IRPs, which bind to IREs in target mRNAs. Hypoxia influences iron metabolism through HIFs which regulate genes such as DMT1, TfR1, and FPN. TfR1 is upregulated in OS, enhancing the activity of the iron-dependent enzyme RRM2 involved in DNA synthesis, as well as promoting activation of the NF-κB signaling pathway via interaction with IKK, supporting cell survival. Red arrows indicate changes in the expression or activity of iron metabolism components in OS compared to normal cells, reflecting increased iron uptake and decreased iron export to support tumor growth and survival.

At the cellular level, iron uptake, storage, and export are tightly regulated through post-transcriptional mechanisms. Central to this regulation are iron-responsive elements (IREs) located in the untranslated regions (UTRs) of mRNAs encoding key proteins involved in iron metabolism. These elements interact with iron regulatory proteins, IRP1 and IRP2, which modulate mRNA stability and translation to maintain cellular and systemic iron homeostasis [30,33]. The activity of IRPs is iron-dependent. Under low intracellular iron conditions, IRPs bind to IREs in the mRNAs of TfR1 and DMT1, stabilizing these transcripts and enhancing iron uptake. Simultaneously, IRP binding to IREs in ferritin and FPN mRNAs represses their translation, reducing iron storage and export. Conversely, under high iron conditions, IRPs dissociate from IREs, leading to destabilization of TfR1 and DMT1 mRNAs, thereby decreasing iron uptake, while allowing for increased translation of ferritin and FPN to promote iron storage and efflux [30,33]. The activity of IRP1 is tightly controlled by the NAD^+^-dependent mitochondrial deacetylase sirtuin 3 (SIRT3), a key regulator of cellular iron homeostasis. Loss of SIRT3 leads to increased production of ROS, which enhances IRP1 binding to IREs and consequently upregulates TfR1 [34]. Emerging evidence also indicates that the expression levels of several miRNAs show a negative correlation with iron availability. In vitro studies have demonstrated that miR-7-5p and miR-141-3p directly target the 3′ IREs of TfR1 mRNA, thereby suppressing TfR1 expression at both the transcript and protein levels [35]. The main pathways involved in cellular iron homeostasis are illustrated in Figure 1. Such regulation is essential for minimizing oxidative stress associated with excessive iron accumulation.

Maintaining systemic iron homeostasis involves a delicate balance between dietary absorption, recycling, storage, and controlled release. While only 1–2 mg of iron is absorbed daily through enterocytes, the majority of iron (20–25 mg/day) is recycled by macrophages, which break down senescent red blood cells to supply iron for erythropoiesis. Excess iron is stored in the liver and macrophages as a mobilizable reserve [36]. At the systemic level, iron homeostasis is primarily regulated by HEPC, a central iron-sensing hormone. HEPC binds to FPN triggering its internalization and degradation and thereby reducing iron release from enterocytes, macrophages, and hepatocytes into circulation [37,38]. This mechanism allows HEPC to act as a key negative regulator of dietary iron absorption and iron mobilization from storage sites.

HEPC expression is finely tuned in response to systemic iron status, inflammation, hypoxia, and erythropoietic demand. Elevated iron and pro-inflammatory cytokines (e.g., IL-6) induce HEPC, while anemia, hypoxia, increased erythropoiesis, and testosterone suppress its production [36]. The bone morphogenetic protein (BMP)-SMAD pathway, particularly involving BMP2 and BMP6 secreted by hepatic sinusoidal endothelial cells, plays a central role in hepcidin transcriptional regulation. Most major signals that influence hepcidin expression, including iron levels, erythropoietic activity, and inflammation, converge on the BMP-SMAD pathway to modulate its transcription [39,40]. Additional regulators include HIFs (especially HIF-2α), which respond to hypoxia by modulating iron metabolism genes such as DMT1, TfR1, and FPN [41,42], and suppressing hepcidin expression even in the presence of inflammatory signals [43] (Figure 1).

Given iron’s essential role in maintaining cellular homeostasis, even minor disruptions in its regulation can have far-reaching consequences. In pathological settings such as cancer, iron metabolism is frequently hijacked to fulfill the increased metabolic and proliferative demands of malignant cells [3,4].

## 4. Iron Metabolism Dysregulation in OS

OS exhibits profound dysregulation of both systemic and cellular iron metabolism. As in many cancers, OS can manipulate systemic homeostatic mechanisms, including inflammatory and neuroendocrine signaling pathways, to reshape the iron landscape in ways that support tumor progression [3,4,5,6,44]. OS patients frequently present with anemia of chronic disease, characterized by reduced iron availability for erythropoiesis despite normal or elevated iron stores [36]. This is largely driven by pro-inflammatory cytokines such as interleukin-6 (IL-6) and tumor necrosis factor-alpha (TNF-α), which can elevate HEPC levels and suppress erythropoietin production [39,45]. The IL-6/STAT3 axis, often hyperactivated in OS [46], can induce HEPC expression, which in turn degrades the iron exporter FPN, thereby reducing serum iron levels and promoting iron retention in macrophages and enterocytes [46,47,48].

HEPC exerts a complex, context-dependent role in cancer biology. Many tumors display dysregulated HEPC expression that supports malignant growth [48,49]. In several cancers, including, breast [50], and ovarian tumors [51], HEPC is upregulated, resulting in reduced FPN levels and iron accumulation, which promotes tumor cell proliferation and survival. Conversely, in hepatocellular carcinoma [52] and certain brain tumors [49], HEPC expression is suppressed, enhancing iron availability through alternative pathways. Notably, a dissociation between systemic and tumor-local HEPC expression has been reported: inflammation may increase circulating HEPC, while tumor cells can locally downregulate it to optimize iron uptake [48,49]. HEPC also modulates the tumor microenvironment, influencing immune cell infiltration and oxidative stress [48,49]. These multifaceted roles make HEPC a potential therapeutic target; however, its modulation must be approached with caution due to its broad physiological impact on iron metabolism and the challenge of balancing systemic and local HEPC levels, which appears to be critical in cancer pathophysiology [48,49].

At the tumor level, OS cells reprogram iron metabolism to meet their high proliferative and metabolic demands. This involves upregulation of iron import systems, such as TfR1, DMT1, and members of the STEAP family, coupled with repression of iron efflux via FPN downregulation (Figure 1). These changes result in enhanced intracellular iron accumulation, which supports key cancer-promoting processes including DNA synthesis, mitochondrial respiration, and cell cycle progression [3,4,16,53,54]. In particular, TfR1 is markedly overexpressed in OS and correlates with higher histological grade, increased metastatic potential, and poor patient prognosis [16,53,54]. Recent studies demonstated that TfR1 promotes OS cell proliferation, migration, and invasion by enhancing intracellular iron uptake and upregulating ribonucleotide reductase M2 (RRM2), a key iron-dependent enzyme subunit required for DNA synthesis. This TfR1-mediated iron accumulation sustains rapid OS tumor growth in vivo [53] (Figure 1).

Beyond iron metabolism and DNA replication, TfR1 has also been implicated in cancer cell survival through the activation of the nuclear factor-kappa B (NF-κB) signaling pathway. By interacting with the inhibitor of NF-κB kinase (IKK), TfR1 promotes NF-κB activation, which in turn inhibits apoptosis and supports tumor cell survival (Figure 1) [54,55]. Iron accumulation in OS cells also contributes to metabolic reprogramming and tumor progression. Elevated intracellular iron levels promote the production of ROS via the Fenton reaction, leading to DNA damage and mutations that support tumorigenesis [56]. In vitro and in vivo studies have shown that iron enhances OS cell proliferation, migration, and invasion, in part by increasing mitochondrial ROS production and driving a metabolic shift known as the Warburg effect, a preference for aerobic glycolysis that supports rapid biosynthesis and growth [57]. This shift is facilitated by the upregulation of mitochondrial iron importers, mitoferrin 1 and 2, which promote iron accumulation in mitochondria and sustain iron-dependent metabolic activity. Importantly, treatment with the iron chelator deferoxamine (DFO) can reverse this metabolic reprogramming in cellular and animal models, highlighting iron’s central role in OS metabolism and its potential as a therapeutic target [57,58]. Experimental evidence from both in vitro and in vivo studies indicates that hypoxia, a hallmark of rapidly growing OS tumors, exacerbates iron dysregulation by stabilizing HIFs. These transcription factors upregulate TfR1 and promote heme degradation via heme oxygenase-1 (HO-1), thereby enhancing intracellular iron levels [59,60,61]. These alterations in iron metabolism are further reinforced at the genetic level. Mutations in TP53, which are frequent in OS, disrupt the tumor-suppressive functions of p53, a key regulator of iron homeostasis [46]. Under normal conditions, p53 downregulates TfR1, promotes the expression of ferritin and HEPC, and suppresses IRPs, mechanisms that collectively reduce intracellular iron availability and inhibit tumor growth [62,63]. In p53-deficient cell models, loss of these regulatory controls results in reduced ferritin and HEPC levels, impaired iron export, and a buildup of labile intracellular iron [64]. This iron overload not only fuels tumor progression by supporting DNA synthesis and metabolic activity, but also contributes to further p53 degradation, creating a vicious cycle that can enhance OS development [4]. Remarkably, iron excess has also been shown to directly destabilize p53 in vitro, exacerbating the loss of tumor suppression and accelerating oncogenesis [4]. Ferritin, the primary iron storage protein, is also altered in OS. Lower expression of the FTL has been associated with shorter metastasis-free survival, while high FTL levels correlate with better treatment outcomes [65]. Together, these systemic and intracellular alterations in iron metabolism highlight the central role of iron in OS progression and underscore its potential as a therapeutic target.

## 5. Manipulating Iron Homeostasis in OS

Understanding iron toxicity, iron dependence, and disruptions in iron homeostasis, including their upstream regulators and downstream effectors, has become an increasingly important focus in cancer research. Given iron’s essential role in supporting tumor growth and survival, its dysregulation represents a therapeutic vulnerability [3,6,66].

As such, targeting iron metabolism has emerged as a promising strategy across various cancer types, including OS [3,67,68]. The following section explores current approaches under investigation that aim to exploit iron dysregulation for therapeutic benefit in OS.

### 5.1. Targeting TfR1 for OS Therapy

Among the various components of iron metabolism, TfR1 serves as a key regulator of cellular iron uptake and homeostasis. As highlighted above, TfR1 is markedly overexpressed in OS cells and plays a critical role in iron uptake, DNA synthesis via RRM2 activation, and tumor cell survival, making it a compelling therapeutic target [16,53,54]. Due to its cell surface accessibility and efficient internalization, TfR1 has been explored in oncology as both a drug delivery vehicle and a direct therapeutic target, as demonstrated in both in vitro and in vivo studies [15,69]. Therapeutic strategies include conjugating TfR1 ligands, such as Tf or anti-TfR1 antibodies, to cytotoxic agents (e.g., chemotherapeutics, proteins, nucleic acids, or nanocarriers), to enable targeted delivery and minimize systemic toxicity [69,70,71,72]. For instance, coupling doxorubicin to transferrin has been shown to enhance tumor specificity and reduce off-target toxicities, such as cardiotoxicity, in breast cancer and leukemia models [73]. Another therapeutic approach involves antagonizing TfR1 function using monoclonal antibodies that block iron uptake, either by inhibiting transferrin binding, preventing receptor internalization and recycling, or promoting TfR1 degradation. These mechanisms lead to iron deprivation, ultimately resulting in tumor cell death [15,58,69,74]. Monoclonal antibodies targeting TfR1 have demonstrated efficacy across several malignancies, including malignant human hematopoietic cells [75], B-cell malignancies [76], and AIDS-related non-Hodgkin lymphoma [77], as shown in both in vitro and in vivo models. In addition to inducing iron starvation-mediated cytotoxicity, these antibodies can also engage the immune system via antibody-dependent cellular cytotoxicity (ADCC), antibody-dependent cellular phagocytosis (ADCP), or complement-dependent cytotoxicity (CDC), thereby enhancing their antitumor activity [15,74,78]. Nevertheless, the widespread expression of TfR1 in all proliferating cells, including hematopoietic precursors, represents a significant limitation to its therapeutic application, due to the risk of off-target effects and systemic toxicity [15]. This highlights the importance of identifying tumor-specific TfR1 targeting strategies or delivery systems to improve selectivity.

Despite this promising rationale, direct TfR1-targeting therapies have not yet been specifically explored in OS. Nevertheless, indirect strategies exploiting TfR1 overexpression have been developed. In one study, TfR1 was used as a target for gene delivery vectors, where transferrin-modified cationic liposomes successfully delivered the p53 gene to human OS cells, leading to significant inhibition of tumor growth both in vitro and in vivo [79]. Thus, although both direct and indirect TfR1-targeted strategies have shown therapeutic promise in other malignancies, their application in OS remains largely unexplored. The marked overexpression of TfR1 in OS underscores the need for further investigation into its potential as a direct therapeutic target.

### 5.2. Iron Chelation for OS Therapy

While TfR1 targeting aims to limit iron acquisition by tumor cells, another complementary approach seeks to reduce intracellular iron availability through chelation. Iron chelators, such as deferoxamine (DFO) and deferasirox (DFX), originally developed to treat iron-overload disorders, have gained attention for their anticancer properties in various malignancies, including leukemia, lymphoma [80], breast [81], colon [82], and gastric cancers [83]. Both agents are FDA-approved and exert antiproliferative effects by depleting intracellular iron, disrupting redox homeostasis, and activating cell death pathways such as apoptosis and autophagy [80,81,82,83]. Interestingly, several studies also report that iron chelators can induce ROS production, which may appear counterintuitive given the role of iron in oxidative stress [14,84]. This effect is thought to involve indirect mechanisms, such as alterations in mitochondrial iron handling, highlighting the complexity of iron chelator activity in cancer cells. Iron depletion has also been shown to decrease the transcription of proteins encoding subunits of the oxidative phosphorylation complexes, resulting in reduced oxidative capacity and elevated ROS levels [85].

In OS, a few preclinical studies have demonstrated the potential efficacy of iron deprivation. Dp44mT, for instance, was shown to suppress proliferation, invasion, and migration of 143B OS cells both in vitro and in vivo [86]. The ability of Dp44mT to redox cycle between Fe^2+^ and Fe^3+^ generates ROS, resulting in oxidative damage of lysosomes and ultimately cell death [86]. More recently, Xue et al. reported that DFO and DFX inhibit proliferation and induce apoptosis in human (MG-63 and MNNG/HOS) and murine (K7M2) OS cells, by disrupting iron homeostasis and activating the MAPK pathway. These effects were associated with increased ROS levels and altered expression of iron-regulatory genes, including upregulation of TfR1 and downregulation of FTH1, FPN, and DMT1 [14]. Based on previous evidence showing that DFO-induced iron-deficient conditions may increase mitochondrial iron and ROS generation in triple-negative MDA-MB-231 breast cancer cells [84], the authors speculated that iron chelators might similarly promote mitochondrial iron accumulation in OS cells, leading to enhanced oxidative stress and ultimately triggering ROS-driven, caspase-dependent apoptosis. Importantly, DFO and DFX appeared well tolerated in animal models, with no major toxicity to vital organs [80,81]. However, contrasting findings have emerged: Argenziano et al. [87] found that iron chelation with DFX or eltrombopag, a thrombopoietin receptor agonist used for thrombocytopenia that also binds and mobilizes iron, either alone or in combination, exhibited iron-chelating activity but did not significantly impair tumor-associated pathways in vitro (143B and MG63 OS cells), including apoptosis, cell cycle progression, proliferation, and ROS production.

The divergent outcomes observed across studies may reflect several factors. First, the intrinsic genetic and metabolic heterogeneity across OS models [88] may be reflected in the differential expression of iron-handling proteins (e.g., ferritin, transferrin receptor, ferroportin), which could in turn affect iron metabolism and chelator sensitivity. Second, microenvironmental factors, including stromal interactions, hypoxia, and oxidative stress gradients, may further modulate drug efficacy [80,89]. Third, pharmacological variability among chelators also plays a role, as these compounds differ in lipophilicity, iron-binding affinity, and subcellular targeting (e.g., cytosolic vs. mitochondrial iron). For example, the enhanced lipophilicity of iron chelators such as DFX and Dp44mT compared with DFO allows them to more readily penetrate the cell membrane [80,90]. Finally, differences in experimental design, such as cell line selection, culture conditions, drug exposure, and iron availability, are also likely to contribute to the inconsistent findings across studies [89]. Overall, while iron chelation holds promise as an adjunctive strategy for OS therapy, the evidence remains preliminary, and further studies are needed to clarify its mechanisms and therapeutic potential in the context of tumor microenvironment interactions.

### 5.3. Ferroptosis as Iron-Related Metabolic Target in OS

Beyond manipulating iron uptake or storage, extensive studies have highlighted the potential to exploit iron-dependent cell death mechanisms as therapeutic opportunities. In this context, ferroptosis has emerged in OS as a distinct form of regulated cell death (RCD) and a notable iron-related metabolic vulnerability [17,66].

RCD is a genetically programmed and tightly controlled process by which cells respond to specific cues or stressors to initiate self-destruction. This mechanism is essential for development, tissue homeostasis, and immune surveillance. However, dysregulation of RCD contributes to various pathological conditions, including cancer, neurodegeneration, infections, and ischemia–reperfusion injury. Among the various RCD modalities, ferroptosis, first described by Dixon et al. in 2012 [91] has emerged as a compelling therapeutic target in cancer, due to its distinct metabolic regulation and potential to modulate tumor behavior [92,93,94,95].

Marked by the buildup of lethal lipid peroxides and iron-driven ROS, ferroptosis is mechanistically distinct from other forms of RCD such as apoptosis and necroptosis, and is closely intertwined with iron metabolism [91,96]. Importantly, ferroptosis inducers, such as erastin, RSL3, and RSL5, cause cell death without displaying the hallmark features of other forms of RCD, such as apoptosis or necroptosis. Conversely, key inhibitors of necroptosis (e.g., RIP1, RIP3) and autophagy (e.g., 3-methyladenine) do not inhibit ferroptosis, reinforcing its mechanistic distinctiveness [91].

This complexity underscores the need for a nuanced understanding of ferroptotic regulation in the tumor microenvironment. Ferroptosis is particularly promising as therapeutic target, especially in tumors that develop resistance to traditional therapies, including evasion of apoptosis [93]. This is highly relevant in OS, a malignancy marked by poor prognosis and high resistance to treatment, where metabolic traits such as iron accumulation [97] lipid peroxidation [98], and redox imbalance [97,98,99] make tumor cells particularly susceptible to ferroptosis-inducing agents [17,100].

Moreover, ongoing research into ferroptosis-related genes (FRGs) and non-coding RNAs (ncRNAs) is uncovering novel biomarkers for prognosis and potential therapeutic targets, enabling patient stratification and paving the way for personalized treatment strategies [67,101,102]. Together, these insights highlight the growing recognition of ferroptosis not only as a unique form of regulated cell death but also as a promising target in clinical oncology, especially for hard-to-treat cancers like OS.

However, it is important to note that in cancer, ferroptosis exhibits a dual role, capable of suppressing tumor growth through oxidative damage, yet under certain conditions, it may also contribute to tumor adaptation and survival by activating stress-adaptive pathways [66,92,103]. Moreover, the release of lipid peroxides and pro-inflammatory mediators from ferroptotic cells can reshape the OS immune microenvironment [21,104,105,106]. While moderate ferroptosis may enhance tumor immunogenicity by promoting dendritic cell activation and CD8^+^ T-cell recruitment, excessive or chronic activation can drive immunosuppression through the accumulation of myeloid-derived suppressor cells (MDSCs), M2-like tumor-associated macrophages (TAMs), and regulatory T cells (Tregs), potentially favoring recurrence or metastasis [104,105,107]. Ferroptosis induction may also impair anti-tumor immunity by affecting effector immune cells, hindering complete tumor eradication [17,67]. This complexity highlights the need for a nuanced understanding of ferroptotic regulation within the OS microenvironment, which is essential for designing strategies that selectively induce ferroptosis in cancer cells while minimizing collateral effects on normal tissues.

#### 5.3.1. Molecular Regulation and Morphological Features of Ferroptosis–General Aspects

At its core, ferroptosis is characterized by the iron-dependent accumulation of lethal lipid peroxides within cellular membranes which begins with a disruption in redox homeostasis, resulting in the unchecked generation of ROS [66,91,96].

ROS, including superoxides and peroxides, are produced through various mechanisms, most notably via the Fenton reaction. These ROS attack polyunsaturated fatty acids (PUFAs) in cell membranes, driving a self-amplifying chain reaction that compromises membrane integrity and leads to cell death. ROS-induced lipid peroxidation is a crucial propulsive step in ferroptosis. The lipid peroxides formed are highly reactive and amplify ROS production, leading to further oxidative damage across cellular compartments. This oxidative cascade overwhelms the cell’s antioxidant defenses, culminating in ferroptotic deat. Mitochondria also contribute to ROS production during oxidative phosphorylation, resulting in oxidative stress, mitochondrial shrinkage, and membrane damage—features typical of ferroptosis [1,24,91,96,108].

Ferroptosis shares signaling pathways and molecular regulators with other forms of cell death. Notably, ROS are central to autophagy and apoptosis, while glutathione (GSH), a key antioxidant in ferroptosis, also contributes to pathways such as cuproptosis, reflecting a complex regulatory interplay [109]. Nevertheless, ferroptosis is marked by specific biochemical and morphological features that set it apart from other cell death modalities.

Destruction and failure of antioxidant mechanisms are crucial in the process of ferroptosis. A key regulatory axis in ferroptosis is the GSH–Glutathione peroxidase 4 (GPX4) antioxidant system. GPX4 is a selenium-dependent enzyme that reduces cytotoxic lipid hydroperoxides (L-OOH) into non-toxic lipid alcohols (L-OH), thereby protecting cells from ferroptosis [110,111]. GPX4 has limited enzymatic activity on its own and relies on the availability of cofactors, such as intracellular GSH, a tripeptide composed of glutamate, cysteine, and glycine, for full functionality. This regulatory axis is critically dependent on cystine uptake, which is mediated by system Xc^−^, a cystine/glutamate antiporter composed of the light chain SLC7A11 and heavy chain SLC3A2 [111,112]. System Xc^−^ imports extracellular cystine in exchange for intracellular glutamate, supplying the cysteine necessary for GSH biosynthesis Inhibition of this antiporter reduces cystine uptake, thereby depleting intracellular GSH levels and compromising GPX4 activity. This reduction in the cell’s antioxidant capacity can result in the accumulation of lipid ROS, thus promoting ferroptosis [110,113,114]. Pharmacological agents such as erastin and RSL3 are commonly used to induce ferroptosis by targeting key components of this pathway. Erastin inhibits system Xc^−^, while RSL3 directly inhibits GPX4. Both compounds disrupt cellular antioxidant defenses, leading to uncontrolled lipid peroxidation and ferroptotic cell death [115].

Emerging evidence also highlights the role of nuclear factor erythroid 2-related factor 2 (Nrf2) as a central transcriptional regulator of antioxidant responses and ferroptosis sensitivity. Under normal conditions, Nrf2 is kept inactive by kelch like ECH associated protein 1 (Keap1)-mediated ubiquitin-proteasome degradation. When cells experience oxidative stress or exposure to toxic compounds, such as under high demand for iron, Nrf2 is released from Keap1, translocates to the nucleus, and activates the expression of various antioxidant and cytoprotective genes [116,117]. In the literature, the expression level of Nrf2 correlates with ferroptosis sensitivity. Cells with downregulated Nrf2 are more susceptible to pro-ferroptotic agents, whereas Nrf2 overexpression prevents both the initiation and execution of ferroptosis, supporting the Nrf2 regulatory network as a viable therapeutic target for managing ferroptosis-related diseases, including cancer and organ injury [116,118].

Lipid metabolism plays a crucial role in regulating lipid toxicity, and its dysregulation is a hallmark of malignancy and a critical driver of ferroptosis [119]. A key enzyme, acyl-CoA synthetase long-chain family member 4 (ACSL4), catalyzes the conversion of long-chain PUFAs, such as arachidonic acid and adrenic acid, into their corresponding acyl-CoA derivatives. These derivatives are then esterified by lysophosphatidylcholine acyltransferase 3 (LPCAT3) and incorporated into membrane phospholipids [120,121].

The PUFA-enriched phospholipids are particularly susceptible to oxidation by ROS and lipoxygenases (ALOXs) or cytochrome P450 reductases (POR), resulting in lipid peroxides that damage membranes and trigger ferroptosis [101,122]. Therefore, the ACSL4-LPCAT3-ALOX/POR pathway may participate in lipid peroxidation-regulated ferroptosis and serve as a biomarker for this process. Notably, loss or inhibition of ACSL4 confers resistance to ferroptosis by limiting the incorporation of PUFAs into membranes [121,122]. Additionally, related studies have shown that the expression levels of ACSL4 and LPCAT3 are upregulated during cellular ferroptosis [123]. More recently, an alternative ferroptosis regulatory mechanism has been identified involving glutathione S-transferase P1 (GSTP1). This cytosolic enzyme catalyzes the detoxification of lipid hydroperoxides to lipid alcohols in a selenium-independent manner [124]. More recently, an alternative ferroptosis regulatory mechanism involving glutathione S-transferase P1 (GSTP1) has been identified in cancer models both in vitro and in vivo. This cytosolic enzyme catalyzes the detoxification of lipid hydroperoxides into lipid alcohols in a selenium-independent manner. GSTP1 has been shown to protect pancreatic cancer cells from radiation-induced ferroptosis [125] and its overexpression is associated with increased cancer risk, particularly in OS [126,127].

In addition to the primary mechanisms described, ferroptosis involves intricate interactions with other cellular systems. The iron–sulfur cluster biosynthesis pathway and mitochondrial iron regulation notably contribute to the labile iron pool and oxidative stress that promote ferroptosis [128]. Other antioxidant defense systems also modulate ferroptosis sensitivity. Among these, coenzyme Q10 (ubiquinone, CoQ10) and ferroptosis suppressor protein 1 (FSP1) have emerged as key modulators in lung cancer cells and OS cells [78,116,117]. Acting independently of GPX4, FSP1 protects against ferroptosis by reducing CoQ10 to its antioxidant form, ubiquinol (CoQH2), in an NAD(P)H-dependent manner. This reaction limits lipid peroxidation and prevents cell death. FSP1 is distributed between the cytosol and the outer mitochondrial membrane and, upon N-myristoylation, translocates to membranes where it exerts its protective effects. Disruption of the FSP1–CoQ10 pathway weakens cellular defenses against oxidative stress, increasing susceptibility to ferroptosis [94,129,130]. Collectively, the molecular signature of ferroptosis includes iron overload, glutathione depletion, GPX4 inactivation, and uncontrolled lipid peroxidation. These interrelated disruptions set ferroptosis apart from other cell death pathways and define its pathophysiological relevance.

In addition to its unique molecular profile, ferroptosis also exhibits distinct morphological features that clearly differentiate it from other forms of RCD [91,95]. One of the most notable characteristics is mitochondrial shrinkage, with mitochondria appearing smaller and more condensed compared to those in healthy or apoptotic cells. This is accompanied by increased mitochondrial membrane density and the loss or complete disappearance of mitochondrial cristae, indicating significant disruption of mitochondrial structure and function [91,95]. In addition, plasma membrane rupture, sometimes with the formation of vesicle-like structures, is observed in ferroptosis, in contrast to the membrane integrity typically maintained during early apoptosis [131,132]. Interestingly, nuclear morphology is largely preserved, with only minor chromatin condensation, distinguishing ferroptosis further from other forms of programmed cell death [131,132].

#### 5.3.2. Ferroptosis-Associated Markers in the Regulation of OS

In OS, ferroptosis appears intricately linked to disease progression and treatment opportunities through a network of underlying mechanisms [17,98,133].

In 2019, Isani et al. [134] were among the first to describe ferroptosis-like cell death in OS. Using Artemisia annua extract on the D-17 OS cell line, they observed iron-dependent, non-apoptotic cell death characterized by a ballooning cellular phenotype, distinct from the nuclear fragmentation typical of apoptosis or necrosis, and accompanied by abnormal iron accumulation [134]. Related studies have highlighted the critical role of the STAT3/Nrf2/GPX4 signaling pathway in regulating ferroptosis and contributing to drug resistance in vitro [118]. STAT3, a well-known oncogenic transcription factor, is frequently activated in OS and is associated with immune evasion, metastasis, and resistance to chemotherapy [135,136]. As a downstream effector of STAT3, Nrf2 promotes the transcription of GPX4, a key enzyme that suppresses ferroptosis by neutralizing lipid peroxides [137]. Notably, Liu and Wang [118] demonstrated that drug-resistant OS cell lines exhibit elevated levels of STAT3, Nrf2, and GPX4 compared to non-resistant lines.

Inhibition of STAT3 led to increased ROS accumulation, reactivation of ferroptosis, and enhanced sensitivity of OS cells to chemotherapeutic agents like cisplatin and 5-fluorouracil. These findings suggest that the STAT3/Nrf2/GPX4 axis acts as a protective mechanism in OS cells against ferroptotic cell death. Importantly, this was the first study to show that targeting the STAT3/Nrf2/GPX4 pathway can sensitize drug-resistant OS cells to chemotherapy by promoting ferroptosis. The use of STAT3 inhibitors or ferroptosis inducers may therefore represent a promising therapeutic strategy to overcome drug resistance in OS [118]. In addition, signaling pathways such as MAPK and HIF, both of which regulate ROS production, have been involved in the regulation of OS development in connection with ferroptosis [138,139]. However, the precise molecular mechanisms underlying this relationship remain largely unclear.

Among the emerging regulators of ferroptosis in OS is GSTP1, acting through its peroxidase activity on lipid hydroperoxides [124,127]. Meta-analyses have linked GSTP1 polymorphisms to OS risk and prognosis. Specifically, GSTP1 variants are associated with increased susceptibility to OS and poorer outcomes in patients undergoing chemotherapy [126,140]. Notably, the GSTP1 null genotype correlates with a higher risk of developing OS. These findings suggest that GSTP1 plays a critical role in ferroptosis regulation in OS and potentially in other bone cell types. As such, targeting the cystine/GSH/GPX4/GSTP1 axis, rather than relying solely on iron-dependent mechanisms, may offer a promising strategy for inducing ferroptosis and improving therapeutic outcomes in OS.

A recently identified GPX4-independent regulatory circuit involves the FSP1-dependent CoQ10 reduction system, which produces ubiquinol, a radical-trapping antioxidant that prevents lipid peroxidation [130]. In their study, Panczyszyn and colleagues demonstrated that OS cells exhibit heterogeneous sensitivity to ferroptosis, which strongly correlates with basal FSP1 expression and the mesenchymal phenotype. Inhibition of FSP1 expression or activity re-sensitized resistant OS cells to ferroptosis. Moreover, the authors identified upstream regulatory mechanisms, showing that NRF2 enhances FSP1 expression upon ferroptosis induction, while p53 contributes to its basal regulation. These findings establish FSP1 as both a predictive biomarker of ferroptosis sensitivity and a potential therapeutic target in OS [130].

Lipid metabolic reprogramming in OS is a complex and dynamic process that plays a crucial role in promoting tumor aggressiveness and resistance to therapy. As thoroughly reviewed by Yin et al. [98], multiple signaling pathways are involved in reshaping lipid metabolism in OS cells, with significant implications for ferroptosis regulation. Key enzymes involved in lipid uptake and de novo lipogenesis, such as ATP-citrate lyase (ACLY) [141], acetyl-CoA synthetase 2 (ACSS2) [142], fatty acid synthase (FASN), and stearoyl-CoA desaturase 1 (SCD1) [143], are frequently upregulated in both in vitro and in vivo models. Their activity not only meets the structural and energetic demands of proliferating cancer cells but also contributes to ferroptosis resistance by modulating lipid composition and redox balance. Additionally, hyperactivation of the mevalonate (MVA) pathway, particularly via 3-hydroxy-3-methylglutaryl-CoA reductase (HMGCR), further supports OS progression and metastatic potential in vivo [144]. These findings suggest that targeting lipid metabolism in conjunction with ferroptosis induction could represent a novel therapeutic strategy. Inhibitors of FASN, statins, and modulators of the MVA pathway have shown potential to disrupt this metabolic rewiring and sensitize OS cells to ferroptotic cell death [145,146]. However, challenges such as compensatory metabolic pathways, acquired drug resistance, and off-target effects may limit the efficacy of such approaches. Despite these limitations, combining lipid metabolism-targeted therapies with conventional treatments may offer promising opportunities to improve outcomes for OS patients.

Beyond metabolic factors, ferroptosis in OS is also regulated at the epigenetic level, adding a further layer of complexity to its modulation [101,147]. Chen and coworkers [148] highlighted the role of the histone demethylase KDM4A, which is found to be overexpressed in OS tissues and associated with poor clinical outcomes. Mechanistically, KDM4A promotes the transcription of SLC7A11, a key component of the system Xc^−^ antiporter, by removing the repressive H3K9me3 histone mark from its promoter region. This epigenetic activation enhances glutathione-dependent antioxidant defenses, thereby stabilizing GPX4 activity and inhibiting ferroptosis. Importantly, silencing KDM4A was shown to downregulate SLC7A11 expression, trigger ferroptosis, and suppress OS cell proliferation and metastasis, thus identifying KDM4A as a potential oncogenic epigenetic regulator and a promising therapeutic target in OS [148].

Ferroptosis not only operates as an independent form of RCD but also shows significant crosstalk with other RCD pathways in a variety of malignancies, including OS [149,150]. For example, Fu et al. [138] developed an ultrasound-activatable nanomedicine for OS that inhibits tumor growth by simultaneously inducing ferroptosis and apoptosis, demonstrating a synergistic effect between the two pathways. Similarly, Lv et al. [139] showed that treatment of murine OS cells with phenethyl isothiocyanate (PEITC) activated multiple RCD pathways, including ferroptosis, apoptosis, and autophagy. Inhibition experiments revealed that while blocking apoptosis or autophagy only partially restored cell survival, the use of a ferroptosis inhibitor almost completely rescued the cells. These findings suggest that ferroptosis may play a dominant role in mediating cell death when co-activated with other RCD mechanisms in OS.

#### 5.3.3. Ferroptosis Inducers in OS

A growing body of evidence highlights the therapeutic promise of ferroptosis inducers in OS. Numerous pharmacological agents and natural compounds have been identified that trigger ferroptosis in OS cells through diverse mechanisms, including inhibition of GPX4 and system Xc^−^, promotion of intracellular iron accumulation, and induction of ROS. These agents disrupt the redox balance and exploit intrinsic vulnerabilities in OS related to iron handling and antioxidant defense, ultimately leading to selective tumor cell death [17,18,151]. The fact that ferroptosis can coexist and interact with other forms of regulated cell death further enhances the therapeutic potential of ferroptosis inducers [17,139]. This section outlines key natural and synthetic ferroptosis inducers in OS, including recently identified agents, and examines their mechanisms of action within the broader context of iron metabolism-targeted therapy.

##### Natural Products Inducing Ferroptosis

Natural products remain a rich source of novel bioactive compounds for cancer therapy, including OS. Dietary supplements and phytochemicals, valued for their anticancer potential and relatively low toxicity to normal tissues, have gained attention as promising therapeutic candidates [152]. Notably, several of these natural compounds have demonstrated potent ferroptosis-inducing activity in OS models, highlighting their potential in iron-dependent anti-tumor strategies [153,154,155].

**Figure 2:**
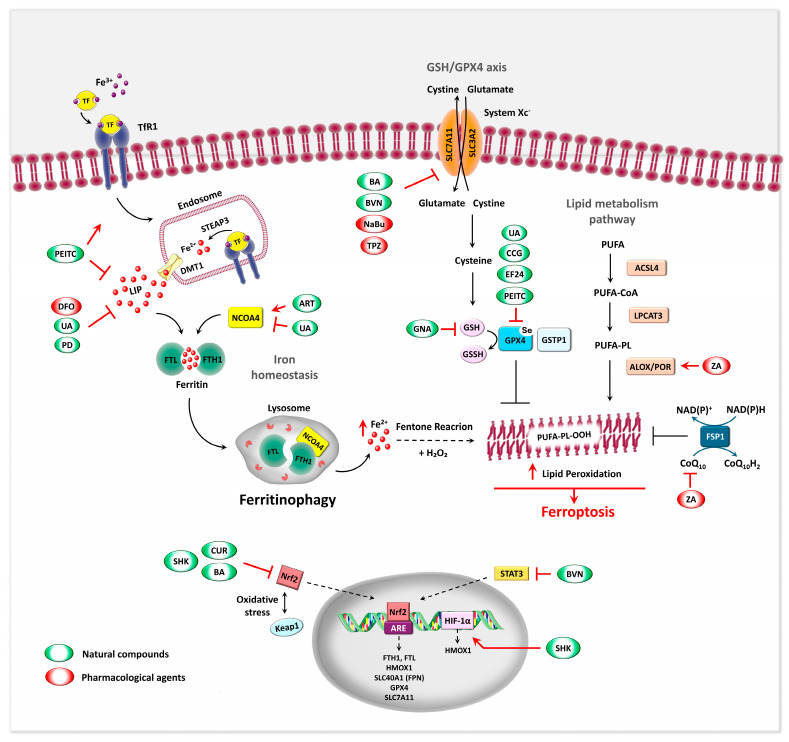
Mechanisms of ferroptosis induction in OS by pharmacological and natural compounds. This figure summarizes key regulatory pathways involved in ferroptosis in OS, including the System Xc^−^/GSH/GPX4 antioxidant axis, iron metabolism and ferritinophagy, and lipid peroxidation. Natural compounds and pharmacological agents modulate these pathways to promote ferroptotic cell death. BVN, BA, NaBu, and TPZ inhibit SLC7A11, impairing GSH synthesis and GPX4 activity. UA, CCG, EF24 and PEITC, weaken antioxidant defenses by suppressing GPX4 activity. UA, PD and PEITC promote iron accumulation through upregulation of TfR1 or DMT1, while ART and UA modulate NCOA4-mediated ferritinophagy, affecting iron homeostasis. Lipid peroxidation is amplified by drugs like ZA, which upregulates POR. Additionally, regulators such as CUR, BA, and SHK modulate oxidative stress and transcription factors (e.g., Nrf2, HIF-1α, STAT3), influencing genes involved in iron and GSH metabolism, further sensitizing cells to ferroptosis. Overall, these agents converge on disrupting redox balance, promoting lipid ROS accumulation, and triggering ferroptosis in OS models. Arrowhead indicates activation; bar-ended line indicates inhibition.

Bavachin (BVN), a flavonoid extracted from *Psoralea*, promotes ferroptosis in OS cell lines by elevating intracellular ferrous iron Fe^2+^, ROS, and MDA levels, effects that are reversed by the iron chelator DFO and ferroptosis inhibitors such as ferrostatin-1 (Fer-1), liproxstatin-1 (Lip-1), and vitamin E (Vit E). BVN’s action is mediated through downregulation of SLC7A11, following STAT3 inhibition and P53 upregulation [155]. Supporting this, STAT3 inactivation has been shown to impair the Nrf2–GPX4 axis, thereby sensitizing OS cells to cisplatin-induced ferroptosis [118]. Similarly, baicalin (BA), a flavonoid derived from *Scutellaria baicalensis*, exhibits potent antitumor activity both in vitro and in vivo [156]. BA directly interacts with Nrf2, a master regulator of ferroptosis resistance, promoting its degradation via the ubiquitin–proteasome pathway. This downregulation of Nrf2 leads to decreased expression of its key downstream targets, GPX4 and the xCT, ultimately triggering ferroptosis [156] (Figure 2, Table 1).

Curcumin (CUR), the principal polyphenol in *Curcuma longa*, has also demonstrated anti-OS potential by promoting ferroptosis. Beyond its known ability to enhance ROS generation and induce apoptosis [157,158], CUR was shown to modulate the Nrf2/GPX4 axis, reducing cell viability, migration, and invasion while increasing apoptosis and ferroptosis in OS cells and xenograft models [159]. These effects were reversed by liproxstatin-1 and bardoxolone methyl (an Nrf2 activator), confirming ferroptosis as the primary mechanism. Expanding on this, the synthetic CUR analog EF24, initially developed for its ROS-modulating and NF-κB-inhibiting properties [160,161] has also been identified as a potent ferroptosis inducer in human OS cell lines. EF24 upregulates heme oxygenase 1 gene (HMOX1), leading to GPX4 suppression, increased intracellular iron, and lipid peroxidation, effects reversible only by ferroptosis inhibitors [154] (Figure 2, Table 1).

**Table 1:** Natural and pharmaceutical agents inducing ferroptosis and ferritinophagy in OS. Combined treatments may include ferroptosis inducers such as erastin; ferroptosis inhibitors: Ferrostatin-1 (Fer-1) and Liproxstatin-1 (Lip-1), autophagy inhibitors: 3-Methyladenine (3-MA) and Bafilomycin A1 (Baf-A1); the necroptosis inhibitor Necrostatin-1 (Nec-1); the apoptosis inhibitor Z-VAD-FMK; antioxidants like N-acetyl-L-cysteine (NAC); the Nrf2 activator Bardoxolone methyl (BM); iron chelators such as deferoxamine (DFO); and standard chemotherapy agents like cisplatin (CIS) and doxorubicin (DOX). Other abbreviations: mitochondrial reactive oxygen species (MitoROS), lipid peroxidation (LPO). ↑: activation or upregulation; ↓: inhibition or downregulation.

Compound	Cell Line/In Vivo Model	Concentrations	Combined Treatment	Molecular Mechanism	Observed Effects	References
Bavachin	MG63, HOS	5–80 μM	DFO, Fer-1, Lip-1, Vit E	↓ SLC7A11 via STAT3 inhibition and P53 upregulation	↑ Fe^2+^, ROS, LPO, ferroptosis	[155]
Baicalin	MG63, 143B/ xenograft	60–120 μg/mL 200 mg/kg/day	Fer-1	↓ Nrf2 ↓ xCT GPX4 axis	↑ ferroptosis, ↓ tumor growth	[156]
Curcumin	MG63, MNNG/HOS /xenograft	22.5 μM	Lip-1, BM	↓ Nrf2 ↓ GPX4	↑ ROS, ferroptosis, apoptosis ↓ tumor growth	[159]
EF24	U2OS, SaOS-2	0.75–1.5 μM	Fer-1	↑ HMOX1 ↓ GPX4	↑ Fe^2+^, ROS, LPO ferroptosis	[154]
Gambogenic Acid	HOS, 143B/ xenograft	0.25–8 μM 30 mg/kg/day	Fer-1, Eastin, NAC	↑ P53, ↓SLC7A11, GPX4	↓ GSH, ↑ ROS, Mito dysfunction, ferroptosis and apoptosis ↓ tumor growth	[162]
β-Phenethyl isothiocyanate	MNNG/HOS, U-2OS, MG-63, 143B/xenograft	30 μM 30 mg/kg/day	z-VAD-FMK, Ner-1, Fer-1, Lip-1, Baf-A1, 3-MA, NAC	↑ TfR1, ↓ FTH1/FPN/DMT1 via MAPK, ↓ GPX4	↑ ferroptosis, apoptosis, autophagy ↓ tumor growth	[139]
Artesunate	MG63, 143B/ xenograft	5–100 μM/ 200 mg/kg/day	Fer-1, DFO, 3-MA, Nec-1, NAC	↑ TFR, DMT1, NCOA4, Mfrn2 ↓ GPX4, xCT	↓ GSH, ↑ Fe^2+^, LPO ferroptosis, ferritinophagy, apoptosis ↓ tumor growth	[18]
Ursolic Acid	HOS, 143B/ xenograft	35 μmol/L	DFO, CIS (20 μmol/L)	↑ TFR, ↓ NCOA4, GPX4	↑ Fe^2+^, LPO ferroptosis, ferritinophagy, ↓ drug resistance ↓ tumor growth	[163]
Shikonin	MG63, HOS/ xenograft	1 or 4 μM/ 2 mg/kg/day	Fer-1, DFO	↑ HIF-1α/HO-1 → mito ROS	↑ Fe^2+^, ROS, LPO, ferroptosis ↓tumor growth	[164]
	MG63, 143B	0.25–10 μM	Fer-1	↓ Nrf2 ↓ xCT GPX4 axis	↑ Fe^2+^, ROS, LPO, ferroptosis	[165]
Polydatin	SAOS-2, U2OS	25–200 μM	Fer-1, NAC, DOX, CIS (10–20 µM)	¯	↑ Fe^2+^, ROS, LPO, ↓ GSH, drug resistance	[151]
Curculigoside	HOS, U2OS, sjsa1, 143b/xenograft and mini-PDX	50 and 75 μM/mL	Fer-1, DFO	↑ TFR, ↓ GPX4, ↑ NF-κB, iNOS in macrophages	↑ Fe^2+^, ROS, LPO, ferroptosis, apoptosis, maturation of RAW264.7 cells	[166]
Tirapazamine	143B, MNNG/HOS, U2OS	5–20 μM	Fer-1	↓ SLC7A11, GPX4	↑ Fe^2+^, ROS, ferroptosis under hypoxia	[167]
Sodium Butyrate	MNNG/HOS, U-2OS/xenograft	0.5–2.5 mM	erastin	↑ ATF3 ↓ SLC7A11	↑ LPO, erastin-induced ferroptosis, ↓ tumor growth	[168]
Zoledronic Acid	MG63, 143B/ xenograft	2–16 μM/100 μg/kg	Lip-1, Z-VAD-FMK, Ner-1	↑ POR	↑ ferroptosis ↓ tumor growth	[169]
	U2OS, MNNG/HOS	1–80 μM	Fer-1	↑ HMOX1, ↓ CoQ10	↑ LPO, ROS, ferroptosis	[170]

Another promising natural agent is gambogenic acid (GNA), a xanthonoid derived from *Garcinia hanburyi* tree. GNA triggers multiple cell death modalities, including ferroptosis and apoptosis in human OS cells by disrupting iron metabolism and oxidative balance. Specifically, GNA modulates the P53/SLC7A11/GSH/GPX4 axis, resulting in GSH depletion, elevated ROS, and mitochondrial dysfunction, with in vivo studies confirming its efficacy in suppressing tumor growth [162]. β-Phenethyl isothiocyanate (PEITC), a naturally occurring isothiocyanate found in cruciferous vegetables, is characterized by favorable pharmacokinetic properties, including low clearance and high bioavailability [171].

In the context of OS, evidence suggests that PEITC may exert its antitumor effects, at least in part, by inducing ferroptosis through disruption of iron homeostasis [172]. In human OS cell lines, PEITC has been shown to alter iron metabolism by upregulating TfR1 and downregulating FTH1, FPN, and DMT1, changes potentially linked to the activation of the MAPK signaling pathway [173]. This disturbance contributes to elevated oxidative stress and weakened redox defense [173]. These findings were further supported in an orthotopic syngeneic OS mouse model, where PEITC treatment significantly reduced tumor size and weight, coinciding with elevated TfR1 and decreased GPX4 expression [139]. Artemisinin, derived from the Artemisia annua plant, and its semisynthetic derivatives are well-known antimalarials that also exhibit promising anticancer properties [174,175], including the ability to modulate key signaling pathways involved in OS proliferation and metastasis [176,177]. Preliminary findings by Isani et al. [134], suggest that artemisinin treatment in canine OS cells may increase the labile redox-active iron pool, potentially leading to iron-driven lipid peroxidation and ferroptosis activation. More definitively, artesunate (ART), an FDA-approved artemisinin derivative, demonstrates anti-OS activity in vitro and in vivo OS models [18]. It upregulated TFR and DMT1, triggering ferritinophagy via NCOA4 upregulation, which increased Fe^2+^ accumulation and initiated ferroptosis. This cytoplasmic iron further activates mitoferrin 2 (Mfrn2)-mediated transport of iron into the mitochondria, resulting in mitochondrial iron overload, lipid peroxidation, and ferroptosis [18]. Other natural compounds contribute to ferroptosis induction in OS through distinct pathways but often converging mechanisms. Ursolic acid (UA), a triterpenoid from kiwifruit, enhances the effects of cisplatin (CIS) by promoting autophagy-mediated ferritin degradation, leading to increased intracellular free iron and lipid peroxidation in OS cell lines and xenografts. Co-treatment with UA and CIS has been shown to downregulate NCOA4 and GPX4, disrupting iron homeostasis and weakening antioxidant defenses, ultimately promoting ferroptotic cell death [163].

Shikonin (SHK), a naphthoquinone derivative extracted from Lithospermum erythrorhizon, induces ferroptosis by activating the HIF-1α/HO-1 axis, a pathway tightly linked to mitochondrial ROS and hypoxia adaptation. SHK-mediated ferroptosis was validated in multiple OS models, highlighting the role of mitochondrial oxidative stress in this process [164]. Mechanistically, SHK can also directly interact with Nrf2, promoting its ubiquitination and proteasomal degradation. This, in turn, downregulates the xCT/GPX4 axis, further compromising antioxidant defenses and enhancing ferroptotic sensitivity [165]. Our group has recently demonstrated that polydatin (PD), a resveratrol glucoside from *Polygonum cuspidatum*, induces redox-dependent cytotoxicity in OS cell lines under both normoxic and hypoxic conditions [151]. PD causes ROS accumulation, GSH depletion, and iron-dependent lipid peroxidation, with minimal cytotoxicity in non-tumorigenic hFOB cells, highlighting its potential therapeutic window. These effects were reversed by ferrostatin-1, implicating ferroptosis in PD-induced cell death. Notably, PD enhances OS cell sensitivity to doxorubicin (DOX) and cisplatin (CIS), which are known to cause metabolic alterations leading to endogenous ROS production and ferroptosis [178]. Further expanding this landscape, curculigoside (CCG), a phenolic compound from the rhizome of *Curculigo orchioides*, has recently been reported to exert significant antitumor effects in OS models. In vitro studies across four OS cell lines demonstrated that CCG not only induces apoptosis but also promotes ferroptosis by enhancing ROS production and altering intracellular Fe^2+^ levels. These effects are accompanied by NF-κB activation and upregulation of iNOS in macrophages, suggesting a link between inflammatory signaling and ferroptotic cell death. The antineoplastic potential of CCG has been further confirmed in vivo, supporting its relevance as an adjuvant agent in ferroptosis-based therapeutic strategies [166] (Figure 2, Table 1).

Despite the promising antitumor potential of natural compounds capable of inducing ferroptosis in OS models, their clinical translation remains highly challenging. To date, all studies have been conducted in preclinical settings, using established OS cell lines or murine xenograft models, primarily aimed at assessing anti-tumor efficacy and in vivo biosafety. While these investigations provide valuable proof-of-concept data and an important foundation for future therapeutic innovations, their translational relevance is limited. A major limitation lies in the poor systemic bioavailability, rapid metabolism, and the frequent use of supra-physiological concentrations of these compounds in vitro, levels that are difficult to achieve or maintain in human tissues through conventional administration [153,179,180]. Moreover, their interactions with other natural compounds in a diet may hinder or complicate consistency of their efficacy [181]. To address these challenges, ongoing research is increasingly focused on the design of long-acting analogs and advanced drug delivery strategies aimed at improving the pharmacodynamics and pharmacokinetics of phytochemicals in human cancer models [182,183,184].

##### Pharmacological Agents Inducing Ferroptosis

Several synthetic and pharmacological agents have also demonstrated ferroptosis-inducing capabilities in OS. Tirapazamine (TPZ), a compound belonging to the benzotriazine class, is a novel hypoxia-activated prodrug that selectively targets and kills hypoxic cells, showing significant promise in tumor treatment [185,186] TPZ has frequently been delivered using nanoplatforms and combined with phototherapy to exploit its hypoxia-activated prodrug properties [187,188]. Shi et al. have reported that TPZ effectively suppresses the proliferation and migration of human OS cells, under hypoxia by inducing ferroptosis [167]. This is evidenced by increased intracellular Fe^2+^ and lipid peroxidation, linked to partial downregulation of SLC7A11, decreased GPX4 levels, and increased ROS [167] (Figure 2, Table 1). Sodium butyrate (NaBu), a short-chain fatty acid and histone deacetylase (HDAC) inhibitor, has been shown to inhibit cancer cell proliferation and induce apoptosis across various cancer types [189]. NaBu was proved to promote the sensitivity of OS cells to erastin-induced ferroptosis by upregulating the activating transcription factor 3 (ATF3) expression, a transcriptional repressor induced during inflammation and infection [190], which subsequently suppresses SLC7A11 transcription [168]. In vivo, the combination of NaBu and erastin significantly reduces tumor growth and increases lipid peroxidation, with the antitumor effect attributed to ATF3-mediated downregulation of SLC7A11 [168].

Among pharmacological agents, zoledronic acid (ZA) is a widely used bisphosphonate for bone metastases from solid tumors [191,192]. Beyond its established clinical use, ZA has demonstrated antitumor effects in OS in several preclinical studies [193,194]. Notably, treatment of OS with ZA, both in vitro and in vivo, has been reported to dramatically increased cell death by upregulating POR, a key driver of ferroptosis, an effect reversible by Lip-1 [169]. POR knockdown significantly attenuated ZA-induced cytotoxicity and suppressed ferroptosis, further establishing POR as a key mediator in this pathway [169]. Furthermore, ZA promotes ferroptosis by depleting intracellular CoQ10, a major lipid-soluble antioxidant and ferroptosis suppressor, and by upregulating HMOX1 expression [170]. These multifaceted actions underscore ZA’s potential as an adjunct in OS therapy by exploiting vulnerabilities in redox metabolism and ferroptotic signaling (Figure 2, Table 1). Importantly, among synthetic ferroptosis-inducing agents, ZA is the only one evaluated in a clinical trial. In the randomized phase 3 OS2006 trial, ZA was administered in combination with chemotherapy and surgery to children and adults with OS to assess possible improvements in survival. However, the addition of ZA to standard chemotherapy did not improve survival outcomes, thus limiting its clinical impact [195]. This underscores the significant translational gap between preclinical ferroptosis research and clinical implementation in OS.

Collectively, the studies presented underscore the potential of ferroptosis induction as a therapeutic strategy in OS. The majority of these natural and synthetic ferroptosis inducers converge on key targets such as SLC7A11/xCT, GPX4, and redox-sensitive transcription factors like Nrf2 and STAT3. While many studies have focused on SLC7A11, the role of SLC3A2, the heavy chain of system Xc^−^, remains underexplored. Machine learning–based analyses of ferroptosis-related genes have recently identified SLC3A2 as a prognostic marker and a modulator of immune response in OS [196]. Its expression was also downregulated in erastin-treated OS cells, suggesting a potential inverse relationship between SLC3A2 levels and ferroptosis sensitivity. However, the molecular mechanisms underlying this regulation remain unclear and warrant further investigation.

Additionally, the ACSL4–LPCAT3–ALOX/POR lipid peroxidation axis is central to ferroptosis execution. Components of this pathway may also serve as predictive biomarkers and therapeutic targets in ferroptosis-driven cancer therapy [98]. Despite their significance, the regulatory effects of ferroptosis inducers on LPCAT3 and ALOX in OS remain largely unexplored, highlighting a promising direction for future research.

#### 5.3.4. Nanomedicine Strategies to Promote Ferroptosis in OS

Nanomedicine has revolutionized cancer diagnosis and treatment by enabling precise drug delivery and targeted distribution, significantly enhancing therapeutic efficacy and minimizing systemic side effects [197]. Its application has extended to OS, where promising advances have been made in developing nanocarriers such as micelleplexes for the targeted delivery of nucleic acids and chemotherapeutic agents [198]. Recent strategies are harnessing nanotechnology to induce ferroptosis in OS cells by promoting ROS generation, depleting GSH, and inhibiting antioxidant defenses, offering new hope for more effective OS treatments [199,200].

Near-infrared (NIR)-mediated phototherapies, such as photothermal therapy (PTT) and photodynamic therapy (PDT), offer advantages like precision, minimal invasiveness, and ease of use [88,201]. However, due to tumor heterogeneity and resistance mechanisms, single-modality PTT or PDT often fails to fully eliminate tumors [202]. In this regard, Wang et al. [183] developed a theranostic nanoplatform (CI@HSA NPs) to potentiate PDT efficacy in OS by concurrently inducing ferroptosis and alleviating tumor hypoxia, in both in vitro and in vivo models. This platform co-encapsulates capsaicin (CAP), a TRPV1 (transient receptor potential vanilloid 1) channel activator [203], that promotes intracellular Ca^2+^ accumulation and inhibits GPX4, and IR780, a NIR photosensitizer, within human serum albumin nanoparticles. Upon NIR irradiation, this system triggers calcium overload, ROS generation, and downregulation of the Nrf2/GPX4 pathway, promoting ferroptosis. Moreover, CAP reduces oxygen consumption via HIF-1α inhibition, mitigating the hypoxic microenvironment that often limits PDT efficacy. These mechanisms converge through the MAPK and PI3K/AKT signaling pathways. In vivo studies confirmed its tumor-suppressive potential and favorable biosafety profile, supporting the value of ferroptosis-based strategies in enhancing PDT outcomes. Building upon these findings, combining PDT with PTT has further demonstrated enhanced efficacy, as NIR irradiation not only promotes ROS generation but also induces local hyperthermia, two key agents in cancer cell [204]. In a newly published study, ref. [199] designed a novel carrier-free nanomedicine SRF@CuSO4.5H2O@IR780 (CSIR) for synergistic ferroptosis and PTT/PDT in OS. CSIR integrates three functional components: Cu^2+^ ions, which react with the intracellular environment to deplete GSH; IR780, which enhances ROS production under NIR irradiation; and sorafenib (SRF), which inhibits the cystine/glutamate antiporter xCT, blocking GSH biosynthesis. This multifunctional system promotes GSH depletion-induced ferroptosis while simultaneously enhancing PDT and PTT, leading to significant antitumor effects, both in vitro and in vivo (Table 2).

Recent advances in nanomedicine have introduced novel Pyrite (FeS_2_)-based therapeutic strategies for promoting ferroptosis in OS. In an initial study, Li et al. [205] developed high-performance FeS_2_ nanoparticles (FeS2@CP NPs) that synergize PTT and chemodynamic therapy (CDT). These nanoparticles exhibit strong catalytic activity in the Fenton reaction that triggers lipid peroxidation and GSH depletion. When combined with near-infrared region II (NIR-II) laser irradiation, FeS_2_ nanoparticles significantly enhance ROS generation, resulting in effective OS suppression through a dual mechanism of apoptosis and ferroptosis both in vitro and in vivo, with minimal side effects [205].

Building upon this foundation, Zheng et al. [200] advanced the concept by engineering FeS_2_-AIPH@Membrane (FAM), a multifunctional nanoparticle system that integrates PTT, CDT, and thermodynamic therapy (TDT). FAM incorporates FeS_2_ for photothermal conversion and AIPH as a radical initiator, all encapsulated within a tumor cell membrane to enhance homologous targeting and immune evasion. Under NIR irradiation, FAM induces robust OS cell death through ROS production and localized hyperthermia.

In vivo experiments using tumor-bearing mice demonstrated significant tumor reduction without systemic toxicity, underscoring its potential as a safe and effective theranostic platform [200] (Table 2). Mesoporous silica-coated iron oxide nanoparticles loaded with Fin56 (FSR-Fin56) have been recently designed as a therapeutic strategy for OS [206].

Fin56, a newly identified type III ferroptosis inducer, directly promotes the degradation of GPX4 exhibiting potent anti-tumor effects [207]. The FSR-Fin56 nanoplatform enables controlled drug release, and upon NIR irradiation, generates localized hyperthermia that enhances the Fenton reaction. This combined effect significantly induces ferroptosis, particularly in ferroptosis-sensitive tumors such as OS. The therapeutic efficacy and safety of the FSR-Fin56 nanovehicle were validated both in vitro and in vivo [206].

The growing chemoresistance of OS in recent decades has significantly impeded therapeutic progress, underscoring the urgent need for alternative or complementary strategies to enhance the efficacy of existing chemotherapy regimens [8,9]. To address this challenge and promote both ferroptosis and chemosensitivity in OS cells, Lin et al. [184] developed ferroptosis-synergistic nanocomplexes (NCs) using hollow mesoporous Prussian blue (HMPB) nanocubes loaded with eriodictyol, a natural flavonoid with tumor-suppressive activity, and cisplatin [208,209]. These nanocomplexes promote ferroptosis through multiple mechanisms, including direct lipid peroxidation, exogenous iron delivery, GSH depletion, and transcriptional inhibition of GPX4 (Table 2). The strategy significantly enhanced ferroptosis and cisplatin sensitivity in OS cells, both in vitro and in vivo, without causing organ toxicity. This approach offers a promising and safe strategy to improve treatment outcomes in OS [184].

In summary, recent innovations in nanomedicine have opened new avenues for OS therapy by strategically inducing ferroptosis. By disrupting GSH metabolism, boosting ROS, and integrating NIR-triggered therapies, these multifunctional platforms pave the way for safer, more effective, and targeted treatments.

#### 5.3.5. Genetic and RNA Biomarkers of Ferroptosis in the Regulation of OS

Comprehensively investigating the pathogenesis of OS is essential to construct effective prognostic signatures and find potential therapeutic targets for guiding clinical treatment decisions in OS. The development of high-throughput sequencing technologies continues to accelerate the exploration of cancer prognostic models based on sequencing results. Ferroptosis is closely related to the occurrence, progression, and prognosis of OS, as well as its sensitivity to chemotherapy [210]. Therefore, targeting genetic and RNA biomarkers linked to ferroptosis offers a promising new approach for early diagnosis, personalized therapy, and improved survival in OS [67,101]. Advances in genomics and RNA biology are therefore crucial for unlocking this therapeutic potential.

##### Ferroptosis-Related Genes in OS

In recent years, prognostic models for OS, grounded in the analysis of ferroptosis-related genes (FRGs), have been successfully devised [67,211]. However, the exact prognostic significance of FRGs in OS remains only partially understood. Manipulating the FRGs, particularly those that suppress ferroptosis, can sensitize tumor cells to chemotherapy, offering a novel therapeutic strategy. Additionally, FRG-derived molecular signatures have proven useful in identifying novel immunotherapy targets, and modulating the OS tumor microenvironment, contributing to improved patient outcomes [196,211,212]. A comprehensive bioinformatics analysis by Li et al. revealed eight hub genes enriched in ferroptosis- and immune-related functions (*CD3D*, *CD8A*, *CD3E*, *IL2*, *CD2*, *MYH6*, MYH7, and *MYL2*) and constructed transcription factor–microRNA networks to characterize regulatory interactions [211]. Another study by Yang et al. proposed a ferroptosis-based prognostic signature comprising five genes, *MUC1*, *MAP3K5*, *LURAP1L*, *HMOX1*, and *BNIP3*, which effectively stratifies OS patients by survival risk and immunotherapy responsiveness [212]. Notably, *MAP3K5*, *LURAP1L*, *HMOX1*, and *BNIP3* were downregulated in OS cells and showed a negative correlation with risk scores, suggesting protective roles. In contrast, *MUC1* was upregulated and positively associated with risk, indicating a possible tumor-promoting function [212].

Similarly, using machine learning approaches, Huang et al. [196] identified six prognostic FRGs (*ACSL5*, *ATF4*, *CBS*, *CDO1*, *SCD*, and *SLC3A2*), which were found to correlate with immune infiltration profiles in OS. These genes are involved in diverse biological processes relevant to tumor progression. For instance, ACSL5, a mitochondrial enzyme involved in long-chain fatty acid metabolism, has been associated with pro-apoptotic effects [213], while ATF4, a transcription factor highly expressed in OS, contributes to anoikis resistance [214]. CBS, overexpressed in several cancers, participates in hydrogen sulfide biosynthesis [215], and CDO1, frequently silenced by promoter methylation, acts as a tumor suppressor [216]. In addition, SCD, a key enzyme in lipid biosynthesis, is implicated in cancer metabolism and progression [217]. Among the identified FRGs, SLC3A2 emerged as a core gene significantly associated with OS prognosis, potentially influencing tumor progression through modulation of the immune microenvironment [196,218].

Additional research developed and validated a prognostic nomogram incorporating five FRGs—*MT1G*, *G6PD*, *ARNTL*, *BNIP3*, and *SQLE*. This model demonstrated strong predictive accuracy, further reinforcing the clinical relevance of ferroptosis-associated pathways in OS prognosis [219]. MT1G plays a role in maintaining metal homeostasis and inhibiting ferroptosis [220]; G6PD, a central enzyme in the pentose phosphate pathway, is known to promote cancer cell growth and has been proposed as a potential biomarker [221]. ARNTL, involved in circadian rhythm regulation, may influence tumor development, though its role in OS is unclear [222]. BNIP3, through its regulation of autophagy and cell death, influences OS prognosis via ROS signaling [223]. Finally, SQLE, an essential enzyme in cholesterol biosynthesis, acts as an oncogene in several cancers, although its function in OS has yet to be fully elucidated [224]

Interestingly, Ma et al. [225] addressed the less explored link between hypoxia, immunity, and ferroptosis in OS. They developed a prognostic model based on two hypoxia-associated genes, *SLC2A1* and *FBP1*, and examined their correlations with immune infiltration and ferroptosis-related genes. They found that *FBP1* positively correlated with AKR1C1 and ALOX15, whereas *SLC2A1* was negatively associated with AKR1C2, AKR1C1, and ALOX15. These findings suggest that hypoxia-related genes may interact with ferroptotic pathways, offering potential targets for future research [225].

A more recent study by Ding et al. [67,211] developed a prognostic model based on FRGs, integrating transcriptomic data from public databases (TARGET, GTEx, GEO), OS cell lines, and patient tissue samples. Through bioinformatic and experimental validation, four FRGs—*BNIP3*, *G6PD*, *PGD*, and *TGFBR1*—were identified as key regulators of OS progression and ferroptosis sensitivity. Functional assays demonstrated that these genes modulate intracellular ROS levels and affect OS cell proliferation, migration, and invasion. BNIP3, typically downregulated in OS, promotes ferroptosis by increasing intracellular iron and ROS, and is associated with favorable prognosis. In contrast, G6PD and PGD, two NADPH-producing enzymes in the pentose phosphate pathway, are overexpressed in OS and contribute to ferroptosis resistance by maintaining redox homeostasis. TGFBR1, a component of the TGF-β pathway, enhances GPX4 expression via NRF2 activation, thereby suppressing lipid peroxidation and ferroptosis. RNA interference targeting G6PD and TGFBR1 sensitized OS cells to ferroptotic death by increasing ROS accumulation, while indirect upregulation of BNIP3 showed potential to restore ferroptosis sensitivity.

In summary, although the role of specific FRGs in OS progression remains underexplored, recent advances in genomics and high-throughput sequencing technologies have begun to shed light on this area. Some FRGs have already been validated as potential targets for OS therapy through ferroptosis-based immunotherapy and prognosis enhancement [196,211,212]. These findings offer new perspectives for understanding the molecular mechanisms underlying OS progression and improving overall survival.

##### Ferroptosis-Related ncRNA Networks in OS

With advancing insights into biomolecular mechanisms, non-coding RNAs (ncRNAs), including microRNAs (miRNAs), long non-coding RNAs (lncRNAs), and circular RNAs (circRNAs), have emerged as critical regulators in cancer pathogenesis. Due to their unique structures and versatile regulatory role, ncRNAs serve as valuable tumor biomarkers and potential therapeutic targets across various cancer types [101,226].

Functionally, ncRNAs lack protein-coding capacity but regulate gene expression at multiple levels. Both LncRNAs and circRNAs can act as scaffolds, decoys, or molecular sponges, acting as competing endogenous RNAs (ceRNAs). They exert their regulatory effects through interactions with miRNAs, transcription factors, and chromatin-modifying complexes. Through these mechanisms, ncRNAs play key roles in regulating vital cellular processes, including proliferation, apoptosis, invasion, migration, and various forms of regulated cell death, such as ferroptosis [226,227,228,229,230].

In the context of OS, specific ncRNAs have been shown to mediate crosstalk between ferroptosis, metabolic reprogramming, and chemoresistance pathways, thereby unveiling new therapeutic opportunities. Their dysregulated expression is frequently associated with immune response modulation, resistance to chemotherapy, and patient prognosis in OS [101,229,231].

*Ferroptosis-related miRNAs*

MiRNAs are small non-coding RNAs (18–24 nucleotides) that regulate gene expression primarily by binding to the 3′-untranslated region (3′-UTR) of target mRNAs, leading to mRNA degradation or translational suppression. Many miRNAs are aberrantly expressed and contribute to tumor development and progression [226,228]. Recent research has uncovered strong associations between miRNAs, iron-dependent cell death, and clinical outcomes in patients with OS [101].

A ferroptosis-related miRNA (FR-miRNA) signature identified miR-593 and miR-635 as significantly correlated with patient prognosis in OS [232]. Functional assays revealed that miR-635 mimics inhibit OS cell proliferation and migration, while miR-593 overexpression promotes these processes, suggesting a tumor-suppressive role for miR-635 and an oncogenic role for miR-593. Clinically, high miR-593 expression was associated with poor prognosis, whereas elevated miR-635 levels correlated with favorable outcomes in OS patients. The study also found that PRNP and HILPDA, two FRGs associated with miR-635 and miR-593, respectively, were differentially expressed between low-risk and high-risk patient groups. These findings indicate that miR-593 and miR-635 not only modulate ferroptosis-related pathways but also have prognostic and therapeutic relevance in OS [232].

Li et al. [231] demonstrated that miR-206 overexpression in OS cell lines promotes ferroptosis by upregulating Prostaglandin-endoperoxide synthase 2 (PTGS2) and KEAP1, which regulate inflammation and oxidative stress response via Nrf2. Conversely, expression of ferroptosis inhibitors such as SLC7A11, GPX4, Nrf2, and HO-1 was markedly reduced. Silencing miR-206 reversed these effects, confirming its role in promoting ferroptosis.

Additionally, miR-206 overexpression was associated with increased intracellular levels of cytosolic ROS, lipid ROS, iron ions, and MDA, indicating activation of lipid peroxidation pathways [231]. Further evidence indicates that miR-188-3p is downregulated in OS tissues and directly contributes to the regulation of ferroptosis by targeting GPX4.

This interaction impacts OS prognosis, with GPX4 expression found to be negatively correlated with miR-188-3p levels, highlighting its tumor-suppressive role [233]. Strategies centered on miRNA-targeted delivery have shown promising efficacy in cancer treatment and are currently advancing through clinical trials, indicating strong therapeutic potential [234]. Due to their ability to facilitate targeted signal delivery, exosome-derived miRNAs are also being explored as potential diagnostic biomarkers for OS [235].

Jiang et al. used OS-derived exosomes as vectors for the transfer of miR-144-3p which targets Zinc Finger E-box Binding Homeobox 1 (ZEB1), impacting iron homeostasis, mitochondrial function, and glutamine metabolism [236]. Moreover, miR-144-3p enhances ferroptosis by upregulating pro-ferroptosis marker ACSL4 and downregulating the anti-ferroptosis regulators SLC7A11 and GPX4, reinforcing its role as a ferroptosis-inducing factor in OS. Notably, once miR-144-3p levels drop below a critical threshold, further reduction has minimal impact on ferroptosis, suggesting a possible self-protective mechanism in OS cells [236]. Another key player, miR-26b-5p, is significantly upregulated in OS and directly suppresses methionine adenosyltransferase 2A (MAT2A), an enzyme critical for the synthesis of S-adenosylmethionine (SAM) from methionine and ATP.

MAT2A modulates the STAT3/SLC7A11 ferroptosis pathway, and its depletion enhances ferroptosis in vivo, highlighting the miR-26b-5p/MAT2A–STAT3/SLC7A11 axis as a critical regulator of ferroptosis resistance in OS [237]. Beyond influencing OS progression, miRNAs can also modulate chemosensitivity by regulating ferroptosis. He et al. [238] reported that METTL1, a tRNA methyltransferase, is downregulated in OS and acts oncogenically. Its overexpression increases miR-26a-5p, which targets FTH1 and promotes ferroptosis, increasing sensitivity to cisplatin and doxorubicin [238].

Lastly, miR-1287-5p is downregulated in OS but can be upregulated upon iron exposure [239]. It promotes ferroptosis by directly targeting and inhibiting GPX4, leading to reduced cell viability and increased sensitivity to cisplatin. The overexpression of GPX4 reverses these effects, confirming the direct role of miR-1287-5p in regulating ferroptosis and therapeutic response in OS [239]. The regulatory roles of FR-miRNAs are summarized in Table 3. Collectively, these findings underscore the critical role of miRNAs in regulating ferroptosis and shaping the biological behavior of OS. By targeting key regulators such as GPX4, SLC7A11, NRf2, and MAT2A, specific miRNAs can either promote or inhibit ferroptotic cell death. Their dual function as biomarkers and therapeutic agents underscores the potential of miRNA–ferroptosis-targeted strategies in improving OS outcomes.

*Ferroptosis-related lncRNAs*

**Figure 3:**
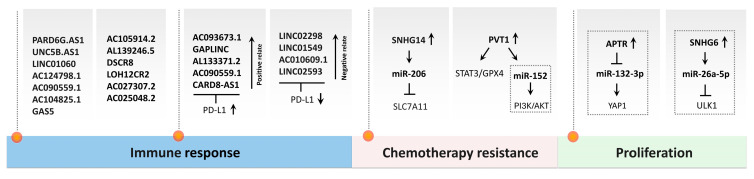
Regulation of OS progression by ferroptosis-related lncRNAs. FRLs influence OS prognosis, immune infiltration, and therapy response. Some lncRNAs are linked to PD-L1 expression and immune evasion, while others drive chemoresistance and ferroptosis suppression via miRNA signaling. LncRNAs with unclear ferroptosis roles are indicated by dashed-line boxes. ↑: activation or upregulation; ↓: downregulation; line ending with a bar indicates inhibition. For further details, see Table 3.

LncRNAs, transcripts longer than 200 nucleotides, are increasingly recognized as key regulators of tumor biology. Although only a small fraction is evolutionarily conserved, functional lncRNAs can act either as tumor suppressors (e.g., MEG3, GAS5) or oncogenes (e.g., HOTAIR, SAMMSON) [101,226,231,246]. In OS, several lncRNAs, including ferroptosis-related lncRNAs (FRLs), have been implicated in critical processes such as immune evasion, drug resistance, and cell death regulation. Numerous studies have highlighted the role of FRLs in modulating immune checkpoint genes and shaping the tumor immune microenvironment [101,102]. For instance, Zhang et al. developed a FRLS-based prognostic model capable of predicting survival outcomes and immunotherapy responses in OS patients [243]. Specific FRLs such as LINC02298, LINC01549, AC010609.1, and LINC02593 showed negative correlations with the expression of the immune checkpoint gene, programmed cell death ligand 1 (PD-L1), suggesting a role in reducing immune suppression, whereas others, including AC093673.1, GAPLINC, AL133371.2, AC090559.1, and CARD8-AS1, were positively associated with PD-L1, possibly contributing to immune evasion [243]. In a related study, Yang et al. identified a set of seven FRLs, comprising three high-risk (APTR, AC105914.2, AL139246.5) and four low-risk (DSCR8, LOH12CR2, AC027307.2, AC025048.2), that significantly influenced OS prognosis and the tumor immune landscape (Figure 3, Table 3). Patients in the FRLnc high-risk group displayed reduced immune cell infiltration and diminished immune functions. Immune checkpoint-related gene expression also varied significantly between the high- and low-risk groups, indicating an immunosuppressive phenotype associated with higher FRL risk scores [102]. Further, Hong-Bin et al. developed a prognostic signature based on iron metabolism-related lncRNAs, including PARD6G.AS1, GAS5, UNC5B.AS1, LINC01060, AC124798.1, AC090559.1, and AC104825.1, which reliably predicts OS patient survival, and immunotherapy responsiveness [227].

Another prognostic model incorporated lncRNAs such as GAS5, UNC5B.AS1, and PVT1, achieving high predictive accuracy [102]. Among these lncRNA, PVT1 has garnered particular attention for its multifaceted oncogenic role across multiple cancers [247].

In OS cells, PVT1 has been shown to suppress ferroptosis by activating the STAT3/GPX4 axis, thereby increasing metastatic potential and treatment resistance. Silencing PVT1 reduced cell proliferation, migration, and invasion, while increasing apoptosis and oxidative stress markers (MDA, Fe^2+^, ROS). These effects were reversed by STAT3 overexpression, further highlighting PVT1’s value as a therapeutic target [240].

Beyond ferroptosis regulation, PVT1 also contributes to chemoresistance. It promotes resistance to gemcitabine, a component of chemotherapy regimens for OS [248] by sponging miR-152 and activating the c-MET/PI3K/AKT signaling cascade [241]. This pathway is also implicated in resistance to standard OS treatments like methotrexate, doxorubicin, and cisplatin [249,250]. PVT1 also promotes glycolysis and tumor growth via the miR-497/hexokinase 2 (HK2) axis, further linking it to metabolic reprogramming in OS [251]. Altogether, these findings indicate that PVT1 is a central regulator of ferroptosis, chemoresistance, and metabolism in OS. Its targeting could offer a promising strategy to restore ferroptosis, reduce tumor aggressiveness, and improve therapeutic responsiveness. Another notable FRL, SNHG14, contributes to resistance against nutlin-3° [231], a p53-MDM2 inhibitor used as an alternative to conventional chemotherapy [231,252].

Li et al. [231], showed that SNHG14 suppresses ferroptosis in nutlin-3a-resistant OS cells via the miR-206/SLC7A11 axis. Knockdown of SNHG14 led to reduced levels of ferroptosis inhibitors (SLC7A11, GPX4), and antioxidants (Nrf2, HO-1), while increasing oxidative stress markers (KEAP1, PTGS2), thereby sensitizing cells to nutlin-3a. These effects were reversed by Ferrostatin-1, confirming the ferroptosis-dependent mechanism [231].

Additionally, FRLs such as SNHG6 and APTR are overexpressed in OS and have been associated with poor prognosis [102,243]. In particular, SNHG6 functions as a ceRNA that promotes proliferation and autophagy via the miR-26a-5p/unc-51-like autophagy activating kinase 1 (ULK1) axis [242]. Although its direct involvement in ferroptosis remains unclear, SNHG6’s inclusion in ferroptosis-related signatures suggests it may indirectly influence redox balance and mediate crosstalk between ferroptosis, autophagy, and apoptosis. APTR, classified as a high-risk lncRNA, contributes to OS progression by suppressing miR-132-3p and upregulating its downstream target yes-associated protein 1 (YAP1), a known oncogene.

Functional assays confirmed that APTR knockdown or miR-132-3p overexpression inhibits proliferation, migration, and invasion while inducing apoptosis, highlighting the oncogenic APTR/miR-132-3p/YAP1 axis [244]. Despite the lack of mechanistic data, APTR’s recurrent presence in FRL-based prognostic models suggests it may act within broader ferroptosis-associated regulatory frameworks (Figure 3, Table 3).

Together, these findings emphasize the critical role of FRLncs as pivotal biomarkers in OS, influencing prognosis, tumor immune landscape, and response to therapy. While the field is still evolving, targeting FRLs presents promising opportunities for synergistic strategies that combine immunotherapy and iron-dependent cell death in OS treatment.

*Ferroptosis-related CircRNAs*

CircRNAs are stable, single-stranded RNA molecules with tissue- and cell-specific expression patterns [253]. Though research on their functions is still emerging, circRNAs show promise as biomarkers in cancer, including OS, due to their stability and regulatory potential as ceRNAs [226,254]. For instance, circRNA_103801 promotes OS cell proliferation and invasion by sponging miR-338-3p and activating the HIF-1/Rap1/PI3K-Akt signaling pathway [255]. Similarly, hsa_circ_0008035 is overexpressed in OS and promotes tumor growth by downregulating miR-375 [256].

**Figure 4:**
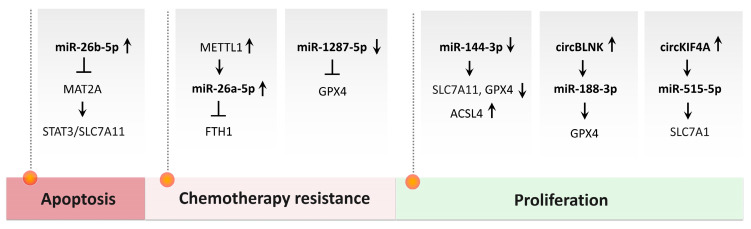
Regulation of OS progression by ferroptosis-related miRNA and circRNA. The diagram illustrates how specific miRNAs and circRNAs modulate ferroptosis, affecting chemotherapy resistance and OS cell survival. Key mechanisms include regulation of FTH1, GPX4, SLC7A11, and ACSL4 via miRNA-circRNA interactions. ↑: activation or upregulation; ↓: downregulation; line ending with a bar indicates inhibition. For further details, see Table 3.

Emerging evidence also suggests a role for circRNAs in ferroptosis regulation within OS. One notable example of a circRNA–miRNA network involved in the occurrence and progression of OS through ferroptosis is represented by circBLNK. As shown by Li et al. [233], circBLNK acts as a ceRNA for miR-188-3p, upregulating GPX4. Knockdown of circBLNK increased MDA, lipid ROS, and mitochondrial superoxide levels, and reduced mitochondrial membrane potential. These ferroptosis-related effects were reversed by GPX4 overexpression, highlighting the circBLNK/miR-188-3p/GPX4 axis as a crucial regulator of ferroptosis in OS [233] (Figure 4, Table 3).

Another example of circRNA-mediated crosstalk with ferroptosis involves circKIF4A, which is also upregulated in OS [245]. He et al. demonstrated that circKIF4A promotes OS cell proliferation and metastasis by sponging miR-515-5p, thereby increasing expression of SLC7A11, a key ferroptosis inhibitor. The circKIF4A/miR-515-5p/SLC7A11 axis contributes to ferroptosis resistance in OS (Figure 4, Table 3). These molecular interactions were validated through luciferase reporter and RIP assays, highlighting this pathway as a novel regulatory pathway linking non-coding RNA networks to ferroptosis suppression in OS [245]. Overall, the emerging role of circRNAs as ferroptosis modulators through axes like circBLNK/miR-188-3p/GPX4 and circKIF4A/miR-515-5p/SLC7A11, adds a new layer of complexity and therapeutic opportunity. Further studies are needed to clarify their mechanisms and clinical applications.

In conclusion, ferroptosis-related ncRNAs, including lncRNAs, circRNAs, and miRNAs, are emerging as key modulators of OS biology. They influence tumor progression, immune evasion, metabolic reprogramming, and therapy resistance, and may serve as points of crosstalk between different forms of cell death and metabolic pathways.

By orchestrating complex regulatory networks involved in redox homeostasis and ferroptotic cell death, these ncRNAs not only show promise as prognostic biomarkers but also represent innovative therapeutic targets. While current findings underscore their potential, further mechanistic studies and clinical validation are essential to translate these insights into effective ferroptosis-based therapeutic strategies aimed at overcoming chemoresistance and improving patient outcomes in OS.

### 5.4. Ferritinophagy: A Novel Target for OS Therapy

In addition to ferroptosis, other iron-regulatory pathways have recently been recognized as potential therapeutic targets. Ferritinophagy is a selective form of autophagy that targets ferritin, the primary intracellular iron storage protein, for lysosomal degradation. This process releases bioavailable iron into the cytosol, supporting essential cellular functions while maintaining iron homeostasis. By regulating the degradation and recycling of stored iron, ferritinophagy plays a critical role in preventing excessive iron accumulation and mitigating iron-induced oxidative damage [257]. This tightly controlled process is orchestrated by the autophagic cargo receptor NCOA4, which mediates the delivery of ferritin to autophagosomes under conditions of elevated cellular iron demand [258]. By mobilizing stored iron, ferritinophagy contributes to essential cellular functions such as heme synthesis and ROS generation [259]. Ferritinophagy plays a fundamental role in various physiological processes, including cellular differentiation, erythropoiesis, and immune responses [257,260].

However, dysregulation of ferritinophagy has been implicated in the pathogenesis of several diseases, including cancer [261,262,263], neurodegenerative diseases [264] and iron overload disorders such as hemochromatosis [265].

In cancer, the phenomenon of “iron addiction”, characterized by increased iron uptake and utilization, renders malignant cells highly dependent on iron-regulatory mechanisms, including ferritinophagy [262,263,266]. Recent studies suggest that enhanced ferritinophagy not only sustains the proliferative and invasive capacity of cancer cells by ensuring iron availability but also sensitizes them to ferroptosis. This dual role highlights ferritinophagy as a double-edged sword in cancer biology: while it supports tumor growth by preserving iron homeostasis, its dysregulation can trigger ferroptotic cell death [262,263]. In OS and other iron-dependent malignancies, ferritinophagy emerges as a novel and exploitable metabolic vulnerability. Therapeutic strategies that modulate this pathway, either by impairing ferritinophagy to disrupt iron metabolism or by enhancing it to promote labile iron accumulation and ROS generation, may sensitize tumor cells to ferroptosis-inducing agents [18,163,267].

#### 5.4.1. Ferritinophagy Regulatory Mechanism

Ferritinophagy is tightly regulated by several mechanisms that govern both the synthesis and degradation of ferritin. A primary regulatory factor is the availability of intracellular iron, which modulates ferritinophagy in response to fluctuations in cellular iron homeostasis. Under normal conditions, this pathway operates at a basal level, but it becomes upregulated during iron deficiency to release iron stored in ferritin, thereby maintaining iron homeostasis [268]. In contrast, during acute iron overload, ferritinophagy is suppressed to favor iron sequestration into ferritin and prevent unnecessary ferritin degradation [269].

A central component of this process is NCOA4, which mediates the trafficking of ferritin and acts as a key regulator of intracellular iron levels [270]. As the rate-limiting factor in ferritinophagy, NCOA4 itself is tightly controlled by intracellular iron concentrations [269]. Ferritinophagy is initiated by the selective binding of NCOA4 to the FTH1 subunit of the ferritin complex [258]. Despite the structural similarity between FTH1 and FTL, NCOA4 specifically interacts only with FTH1 [258,271]. Interestingly, although the molecular details of this interaction were previously unclear, recent cryo-electron microscopy studies have resolved the structure of the NCOA4–FTH1 complex, revealing the binding interface [32]. This structural insight provides a foundation for the rational design of molecules that disrupt the NCOA4–FTH1 interaction, offering new opportunities to therapeutically target ferritinophagy in iron-related disorders. Once bound to FTH1, NCOA4 interacts with LC3–PE on the autophagosome membrane and microtubule-associated proteins, facilitating the selective sequestration of ferritin complexes into autophagosomes for subsequent lysosomal degradation [272]. As NCOA4-mediated ferritinophagy controls cellular free iron levels, its modulation influences sensitivity to ferroptosis. Indeed, downregulation of NCOA4 decreases sensitivity to ferroptosis induction, while upregulation increases it [273].

The interplay between NCOA4-mediated ferritinophagy and ferroptosis is tightly modulated by intracellular iron homeostasis, autophagy-related genes (e.g., ATG3, ATG5, ATG7), and functional lysosomal pathways [274]. NCOA4 activity can also be influenced by DNA damage response elements like ATM, which enhances ferritinophagy via NCOA4 phosphorylation [275] and by signaling pathways such as IL-6/STAT3 and Hippo [276], which modulate NCOA4 expression and activity [277].

**Figure 5:**
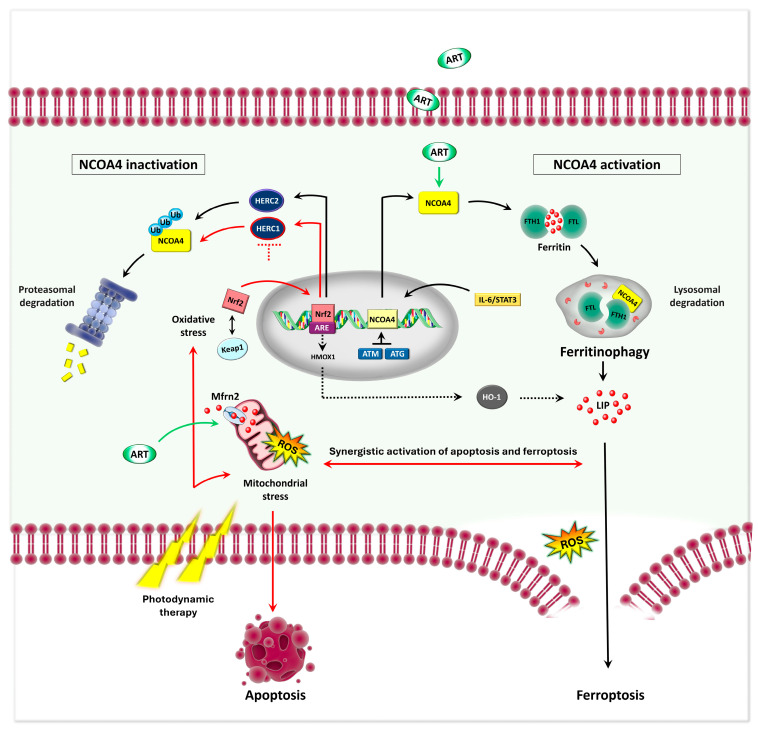
The Ferritinophagy pathway and its modulation in OS. The regulatory mechanisms of ferritinophagy through NCOA4 activation and inactivation, in the absence of induction modulators, are indicated by black arrows. NCOA4 selectively binds the FTH1 subunit of ferritin, directing it to autolysosomes for degradation and iron release. Ferritinophagy can be regulated by ATGs, DNA damage sensors like ATM, and signaling pathways such as IL-6/STAT3. Nrf2 modulates NCOA4 stability via the E3 ligase HERC2, linking oxidative stress responses and iron metabolism. Under oxidative conditions, Nrf2 activates antioxidant gene expression and HO-1-mediated iron release, intersecting with ferritinophagy (dashed black line). Red and green arrows indicate ferritinophagy induction in OS by photodynamic therapy and ART, respectively. ART promotes NCOA4-dependent ferritinophagy and Mfrn2 activation, while photodynamic therapy targets the HERC1–NCOA4 axis to synergistically induce apoptosis and ferroptosis. Under PDT-induced oxidative stress, NRF2-mediated activation of HERC1 enhances NCOA4 degradation, inhibiting ferritinophagy. Dashed red lines indicate that HERC1 inhibition stabilizes NCOA4, restoring ferritinophagy and ferroptotic cell death.

HO-1 is a rate-limiting enzyme responsible for the oxidative degradation of heme, resulting in the release of free iron and playing a key role in mediating cellular ferritinophagy and ferroptosis [278]. The Nrf2/HO-1 pathway intersects critically with autophagy-related pathways: under oxidative stress, Nrf2 translocates to the nucleus to activate antioxidant gene expression, while also regulating HO-1-driven iron release. Moreover, Nrf2 controls the ubiquitin-proteasome degradation of NCOA4 through the E3 ubiquitin ligase HERC2, thereby modulating ferritinophagy and susceptibility to ferroptosis [278,279] (Figure 5).

The interplay of autophagy-related genes (ATG3/5/7), lysosomal function, and Nrf2 signaling underscores the complexity of this regulatory network. In cancer biology, this connection highlights a crucial regulatory node where iron metabolism, redox biology, and programmed cell death converge. Pharmacological or genetic inhibition of NCOA4, autophagy-related pathways, or lysosomal activity has been shown to reduce ferritin degradation and suppress ferroptosis, further emphasizing the essential role of NCOA4 in this axis [260,280,281]. Conversely, strategies that enhance NCOA4-mediated ferritinophagy may sensitize cancer cells to ferroptosis, representing a compelling direction for therapeutic development, particularly in tumors exhibiting ferroptosis resistance or iron addiction, including OS [282,283,284].

#### 5.4.2. Modulation of Ferritinophagy in OS: Emerging Therapeutic Approaches

NCOA4 may play a role in carcinogenesis, including OS, and the intersection of ferritinophagy and ferroptosis pathways may represent a potential therapeutic target. As mentioned above (Section 5.3.3), Huang et al. [18] recently investigated the relationship between ART-induced ferroptosis and autophagy in human OS. In both in vitro and in vivo models, ART was shown to induce NCOA4-mediated ferritinophagy, leading to FTH1 degradation and increased intracellular iron release (Figure 5, Table 1). ART also upregulated TFR and DMT1, elevating Fe^2+^ levels and promoting mitochondrial iron transport via Mfrn2. The resulting mitochondrial iron overload triggered excessive ROS production, lipid peroxidation, and ultimately ferroptosis. In vivo, ART administration significantly inhibited tumor growth through ferroptosis induction. These findings underscore the potential of ART as a therapeutic agent in OS by promoting NCOA4-driven ferritinophagy [18]. Iron metabolism and its cancer-related changes are still poorly understood in veterinary medicine. Early research have shown increased iron uptake and TfR-1 expression in canine OS, suggesting a potential role for iron metabolism in tumor progression and treatment [16,285]. Canine OS are increasingly recognized as a valuable comparative model for human OS, given their similar clinical and molecular characteristics [286]. Building on this, Power et al. [19] investigated the immunohistochemical expression of ferritinophagy markers (NCOA4 and FTH1) and proliferating cell nuclear antigen (PCNA) in normal canine bone and canine OS samples. While normal bone showed minimal expression of these markers, the majority of neoplastic cells in canine OS exhibited strong immunoreactivity for NCOA4, FTH1, and PCNA. These results suggest that canine OS cells may activate ferritinophagy to meet their elevated iron demands.

PDT has also emerged as a promising treatment modality for OS [201]. Although apoptosis is considered the primary mechanism underlying PDT’s therapeutic effect, this approach remains limited by issues such as low efficacy and resistance. Recent studies propose that combining PDT with ferroptosis induction may enhance its effectiveness by boosting ROS levels through the Fenton reaction, as demonstrated in breast cancer models both in vitro and in vivo [287]. In this context, Zhang et al. [267], examined the mechanism by which PDT, specifically using the photosensitizer pyropheophorbide-a methyl ester (MPPα), exerts anti-tumor effects in OS cell lines. Their study revealed a synergistic activation of apoptosis and ferroptosis, and identified HERC1, an E3 ubiquitin ligase, as a key negative regulator of ferritinophagy. Under oxidative stress triggered by MPPα-PDT, HERC1 is activated via the NRF2 signaling pathway, promoting the ubiquitination and degradation of NCOA4, thereby suppressing ferritinophagy. This limits iron release from ferritin and dampens ferroptosis. However, inhibition of HERC1 preserved NCOA4 stability, restored ferritinophagy, and enhanced intracellular iron availability, leading to increased lipid peroxidation and ferroptotic cell death [267] (Figure 5). Together, these findings identify the HERC1–NCOA4 axis as a novel regulatory node that could be targeted to enhance the therapeutic efficacy of PDT, offering a more effective therapeutic strategy against OS. In summary, although current findings on OS are preliminary, targeting ferritinophagy-related pathways, either pharmacologically or through combination strategies like PDT, presents a compelling opportunity to overcome resistance mechanisms and improve outcomes in both human and canine OS.

## 6. Conclusions and Perspectives

The intricate interplay between iron metabolism and OS pathophysiology has emerged as a compelling focus in cancer research. OS cells exhibit a profound dependency on iron to sustain their proliferative and metastatic potential [53,54,57]. Yet, this metabolic reliance also renders them vulnerable to iron-mediated cytotoxicity, particularly through ferroptosis [17,100]. These dual aspects of iron biology, fueling tumor growth while also enabling its suppression, position iron metabolism as a promising but complex therapeutic target.

Among emerging approaches, ferroptosis-based therapies, utilizing traditional Chinese medicine, small-molecule antitumor agents, and nanoparticle-mediated delivery systems, have emerged as particularly promising in preclinical settings [17,184,206,210]. These interventions offer innovative opportunities to overcome chemoresistance and selectively target aggressive, treatment-refractory OS cell populations. Moreover, targeting ferroptosis-related mechanisms such as ferritinophagy [18,267], lipid metabolic reprogramming [98], and the modulation of non-coding RNAs (miRNAs, lncRNAs, and circRNAs) [101,147], further broadens the therapeutic landscape. Integrating these approaches into combinatorial regimens with chemotherapy, radiotherapy, or immunotherapy could amplify treatment efficacy and improve patient outcomes.

Despite this progress, current research remains limited in scope. Most studies focus heavily on the GSH-GPX4 antioxidant axis [155,156,159], while other key regulatory pathways, including NCOA4-mediated ferritinophagy [18], lipid peroxidation [98], and iron-export mechanisms, are comparatively underexplored. Additionally, while ferroptosis has been linked to enhanced responses to chemotherapy, its potential to improve radiotherapy and immunotherapy outcomes in OS remains largely untapped [17]. Importantly, emerging evidence underscores the impact of iron metabolism in shaping the tumor immune microenvironment [13,102,211].

Inducing ferroptosis may inadvertently suppress anti-tumor immunity by fostering an immunosuppressive milieu and harming immune cells [67,288]. Given that most ferroptosis inducers lack cell or tissue selectivity, such off-target toxicity toward immune and stromal populations raises important safety concerns [17,66]. To address these challenges, combinatorial strategies that integrate ferroptosis inducers with immunomodulatory or anti-inflammatory agents could help balance efficacy and safety. Incorporating FRGs and immune-related signatures into predictive models may further elucidate the complex crosstalk between ferroptosis and immunity, facilitating the development of more precise, safer, and selective ferroptosis-based therapies for OS [67,102,288]. Still, several challenges must be addressed. While iron chelators like DFO and DFX, have shown some promise, results are not uniformly consistent across OS models, likely due to the intrinsic heterogeneity of the tumor and its capacity to adapt to iron deprivation [14,87]. Similarly, despite the marked overexpression of TfR1 in OS [53], direct TfR1-targeting approaches remain largely unexplored and warrant deeper investigation.

The context-dependent role of iron in bone biology represents another significant concern. Uncontrolled induction of ferroptosis, while potentially therapeutic in tumor cells, may impair healthy bone regeneration by affecting osteoblasts and stromal precursors, as iron is essential for collagen synthesis and extracellular matrix formation. Excessive iron accumulation can suppress osteoblast activity, leading to diminished bone strength and regenerative capacity [100,289,290]. Recent findings by Smirnova et al. [127] further clarify the differential sensitivity to ferroptosis among OS cells and various mesenchymal stromal cell populations. Specifically, while undifferentiated human bone marrow stromal cells and OS cells are highly prone to ferroptosis, differentiation appears to confer substantial resistance in stromal cell populations. Mechanistically, GPX4 seems to regulate ferroptosis in stromal cells, whereas GSTP1 plays a dominant role in OS cells.

These insights underscore the need for cell-selective targeting strategies to avoid off-target toxicity. Innovative technologies, such as cell-specific nanocarriers and fluorescent reporters of lipid peroxidation, offer promising tools to achieve this precision, enabling more refined therapeutic approaches that maximize efficacy while preserving normal bone function [291].

In summary, targeting iron homeostasis and iron-related cell deaths holds strong therapeutic promise in OS. However, success will depend on the development of context-specific, precision-based therapeutic approaches that account for tumor heterogeneity, iron’s physiological roles, and the complex tumor microenvironment. While preclinical models provide valuable mechanistic insights, some studies suffer from methodological limitations or inconsistencies. Furthermore, the biological and genetic divergence of these models from human tumors highlights the need for standardized and physiologically relevant systems [292,293]. To enhance clinical translatability, future research should focus on developing animal and patient-derived models that more accurately recapitulate OS heterogeneity and microenvironmental dynamics, thereby improving the predictive value of iron-targeted interventions [293,294]. Progress in preclinical modeling, molecular profiling, and precision drug delivery will be crucial to advancing these approaches toward clinical impact.

In this context, principles of synthetic lethality (SL) could provide an additional translational advantage [295]. Targeting metabolic vulnerabilities within iron homeostasis represents a rational SL-informed strategy to induce conditional lethality while minimizing toxicity in normal tissues [295]. Ferroptosis, intrinsically linked to oxidative stress, iron accumulation, and hypoxic metabolic rewiring, aligns closely with the SL framework by exploiting the tumor’s reliance on compensatory survival mechanisms [296]. Thus, combinations of ferroptosis induction, iron chelation, and modulation of compensatory pathways may create context-specific conditional lethality in iron-addicted OS cells, enhancing therapeutic precision and the durability of anti-tumor responses.

## Figures and Tables

**Table 2:** Nanocarrier-based strategies for ferroptosis induction in OS.

Nanoplatform	Cell Line/In Vivo Model	Combined Treatment	Molecular Mechanism	Observed Effects	References
CI@HSA NPs (capsaicin + IR780 in albumin NPs)	143B, HOS xenograft	capsaicin + PDT	↑ TRPV1/Ca^2+^, ↓ Nrf2/GPX4, HIF-1α; ↑ MAPK, ↓ PI3K/AKT	↑ ferroptosis, ↓ tumor hypoxia, ↓ tumor growth, good biosafety	[183]
CSIR (SRF@CuSO_4_·5H_2_O@IR780)	K7M2 xenograft	PDT + PTT	↓ xCT	↓ GSH, ↑ ROS, ↑ ferroptosis, ↓ tumor growth	[199]
FeS2@CP NPs	U2OS, MNNG/HOS xenograft	PTT + CDT	↓ GPX4	↑ Fe^2+^, ROS, LPO, ↓ GSH, ↑ ferroptosis, apoptosis, ↓ tumor growth, minimal side effects	[205]
FAM (FeS2-AIPH@Membrane)	MNNG/HOS xenograft	PTT + CDT + TDT	__	ROS-induced tumor ablation, low systemic toxicity	[200]
SR-Fin56 (Fe_3_O_4_@SiO_2_-RGD loaded with Fin56)	MNNG/HOS xenograft	PTT + CDT	↓ GPX4	↓ GSH, ↑ Fe^2+^, ROS, LPO, ↑ ferroptosis, safe and effective in vivo	[206]
HMPB@Cisplatin@Eriodictyol	U2OS, MG63, 293T, xenograft	cisplatin + eriodictyol	↓ GPX4	↓ GSH, ↑ Fe^2+^, ROS, LPO, ↑ ferroptosis, ↑ cisplatin sensitivity; no organ toxicity	[184]

↑: activation or upregulation; ↓: inhibition or downregulation.

**Table 3:** Regulatory role of ferroptosis-related ncRNA in OS.

FR-miRNAs				
	Expression Trend in OS	Functional Effects	Model Type	References
miR-593	upregulated	regulates FRGs; promotes proliferation and migration; associated with poor prognosis.	HOS, in silico	[232]
miR-635	downregulated	regulates FRGs; suppresses proliferation and migration; high expression correlates with favorable prognosis.	HOS, in silico	[232]
miR-206	upregulated	promotes ferroptosis via ↑ PTGS2, KEAP1 and ↓ GPX4, SLC7A11, Nrf2, HO-1; increases ROS, Fe^2^, LPO; negatively regulated by lncRNA SNHG14	SJSA1	[231]
miR-188-3p	downregulated	promotes ferroptosis via ↓ GPX4T; tumor-suppressive; negatively regulated by circBLNK	HOS, SJSA-1, MG63, U2OS, tissues	[233]
miR-144-3p	downregulated	promotes ferroptosis via ↓ ZEB, ↑ ACSL4, ↓ GPX4, SLC7A11; affects iron homeostasis and metabolism, inhibits proliferation, migration, and invasion of OS cells.	143B, SW1353, MG-63, SaOS-2, U2OS, nude mice	[236]
miR-26b-5p	upregulated	↓ MAT2A → promotes ferroptosis via ↓ STAT3/SLC7A11	MNNG/HOS, U2OS, nude mice	[237]
miR-26a-5p	upregulated	promotes ferroptosis via ↓ FTH1; increases sensitivity to cisplatin/doxorubicin; regulated by METTL1, which enhances miR-26a/FTH1 interaction	U2OS, 143B xenograft	[238]
miR-1287-5p	downregulated	promotes ferroptosis via ↓ GPX4; ↑ cisplatin sensitivity	SaOS2, U2OS	[239]
FR-lncRNAs				
PVT1	upregulated	inhibits ferroptosis via ↑ STAT3/GPX4; ↑ metastasis	MG63	[240]
	upregulated	regulates glycolysis and chemoresistance through ↑ c-MET/PI3K/AKT	MG63	[241]
SNHG14	upregulated	sponges miR-206 → inhibits ferroptosis via ↑ SLC7A11; increases nutlin-3a resistance	nutlin3a-resistant NR-SJSA1	[231]
SNHG6	upregulated	promotes autophagy via miR-26a-5p/ULK1 axis	MG63	[242]
	upregulated	possibly regulates ferroptosis indirectly, correlates to OS prognosis and immunity	in silico	[243]
APTR	upregulated	suppresses miR-132-3p → ↑ YAP1 → promotes proliferation and invasion	MG63, 143B, Saos-2, HOS	[244]
	upregulated	included in high-risk ferroptosis-related signatures	in silico	[243]
GAS5	—	included in iron metabolism-related prognostic models; correlates with OS progression and immunotherapy response.	in silico	[227]
UNC5B-AS1				
LINC01060				
AC124798.1				
AC104825.1				
LINC02298	downregulated	negatively correlated with PD-L1 → reduce immune suppression	in silico	[243]
LINC01549				
AC010609.1				
LINC02593				
AC093673.1	upregulated	positively correlated with PD-L1 → promote immune evasion	in silico	[243]
GAPLINC				
AL133371.2				
CARD8-AS1				
DSCR8	downregulated	correlates with favorable prognosis; involved in immune cell infiltration	in silico	[102]
LOH12CR2				
AC027307.2				
AC025048.2				
FR-circRNAs				
circBLNK	upregulated	sponges miR-188-3p → inhibits ferroptosis via ↑ GPX4; promotes OS progression	HOS, SJSA-1, MG63 and U2OS, tissues	[233]
circKIF4A	upregulated	sponges miR-515-5p → inhibits ferroptosis via ↑ SLC7A11, ↑ metastasis and proliferation	SOSP-9607, HOS, U2OS, SW1353, Saos-2, tissues	[245]

↑: activation or upregulation; ↓: inhibition or downregulation; —: not determined.

## Data Availability

No new data were created or analyzed in this study.
